# An Introduction to the Study of Animal Magnetism

**Published:** 1838-07-01

**Authors:** 


### ANIMAL MAGNETISM.

An Introduction to the Study of Animal Magnetism. By the Baron *Dupotet de Sennevoy*. With an Appendix, containing Reports of British Practitioners in favour of the Science. Small 8vo. pp. 388. London, 1838.

Who can live in this, who can have lived in any age, save those dark ones, when the Goth and the Vandal blotted out the rays of the sun of science in the West, without perceiving the progress of the human mind? The advancing tide of philosophy has had its Canutes, who have planted their feet upon the sand, and forbad its coming. But that tide has rolled, and will still roll on:

Labitur et labetur in omne volubilis ævum.

The history of philosophy teaches us the curious fact, that its progress has been at times assisted by error. A false theory led Columbus to the rectification of geography, and the discoveries of Galileo confuted his own anticipations. Ingenious fallacies may mislead for a season, but, stimulating inquiry and eliciting facts, truth is ultimately generated from the very seeds of falsehood.

It cannot be denied, that the noblest additions to human knowledge have been violently opposed by prejudice and ignorance. Galileo, Harvey, and Jenner, were reviled and persecuted, each in his season and in accordance with the genius of his age. But neither can it be denied, that, if, in these instances, contemporary judgment was erroneous, or rather contemporary prejudice was strong, in many other cases, it has determined well, and posterity have set their seal on the damnation it pronounced. The voice of the public is generally rather right than wrong, and its false awards have been the exception, not the rule.

The temporary condemnation of Galileo and of Harvey has been the ruin of many a fool. If the world laughs at his folly, he consoles himself by thinking that it ridiculed those great men—and, as he *resembles* them in being a butt, he is sure he *will resemble* them in being immortal. The case is a tally of that of some young gentlemen, who deem themselves Byrons, not because they write new Giaours or Harolds, but because they wear their shirt collars open.

But we fear that the English are a stiff-necked people. Sir Thomas More tells us that Raphael Hythloday, the Utopian traveller, found, in England, all proposals for improvement encountered by the remark, that—"'Such things pleased our ancestors, and it were well for us if we could but match them;' as if it were a great mischief that any should be found wiser than his ancestors." The curse of the national character is its practical temper. It is much too exact, too matter of fact. A Laputan savant, would, we fear, find it useless to propose extracting sun-beams out of cucumbers here.

On this account we feel timid with respect to the success of the science, which has received the name of Animal Magnetism. It is almost too etherial for this Bœotian atmosphere, and its mysticisms and sublimities can scarcely submit to our gross arithmetic computations. The proselytes to animal magnetism must be imbued with something of the spirit of its founder. They must entertain a due contempt for the insignificance of what in our grovelling spirit we consider 'exact reasoning' and 'demonstration;' must possess a strong taste, and profess a ready belief for the subtle essences and mystical powers of the elements.

The great Mesmer, the immortal discoverer of this science, was, as he ought to be, a man of quick perception and "vivid imagination." He started by maintaining, in a thesis, the influence of the planets on the human body —a doctrine which, like magnetism, appearing to be contrary to common sense, has been most injuriously neglected. Now that magnetism is taking its proper place among the sciences, it may be hoped that planetary influence will also have a place beside it. When men are magnetised, children should have their horoscopes. We shall at length, perhaps, do justice to the shades of Sidrophel and Lilly. Like Mesmer, those illustrious men—

"With the moon were more familiar
Than e'er was almanack well-willer;

Knew when she was in fittest mood
For cutting corns or letting blood;
When for anointing scabs or itches,
Or to the bum applying leeches."

But to return to the kindred spirit of Mesmer. He maintained, says M. Dupotet, that the sun, moon, and fixed stars, mutually affect each other in their orbits; that they cause and direct on our earth a flux and reflux, not only in the sea, but in the atmosphere; and affect in a similar manner all organised bodies, through the medium of a subtile and mobile fluid, which he conceived pervaded the universe, and associated all things together in mutual intercourse and harmony. How analogous is the simple majesty of this doctrine, with that of animal magnetism. How independent of the debasing spirit of demonstration.

"About this time the mineral magnet was held in much repute as a therapeutical agent, and Father Hell, a Jesuit, and professor of astronomy at Vienna, invented steel-plates of a peculiar form, which he applied with much success in the treatment of various diseases. The medicinal efficacy of these mineral-plates he imagined depended on their form; but Mesmer soon discovered that although their application apparently produced certain manifest effects, yet that he could, without using them at all, by passing his hands from the head downwards towards the feet of the patient, even at a certain distance from the body, induce the same effects." 7.

The powers of the mineral magnet over the ills to which flesh is heir, are so familiar to the medical world, that there could not be culled a more apt illustration of the equally marvellous and equally certain powers of magnetism of the animal sort.

It is pleasant to contrast the good and the bad, and it is useful to signalize the merits of the former. While the prejudiced Académie des Sciences de Berlin, replied to an address of Mesmer's:—"Qu'il n'était qu'un visionnaire;" the more philosophical Académie of Bavaria elected him a member.

Mesmer's career was a brilliant one. Every where he effected cures—no where, does M. Dupotet tell us that he failed. The stupid Academy of Paris was, of course, opposed to him. It could not be expected that a society composed of the best medical science of France, could be otherwise than prejudiced, jealous, and blind. As the acmé of its mingled folly and injustice, a Commission of the Academy had the absurdity to declare that:—"L'imagination fait tout, le magnetisme animal est nul: imagination, imitation, attouchement, telles sont les vraies causes des effets attribués au magnétisme animal." But though the Academy stultified itself in this manner, the true love of science, and candour, and conviction, were not extinct in France. A few doctors, and crowds of "persons of rank and fortune," saw, believed, and were initiated. "Sociétés de l'Harmonie" were every where instituted, and the progress of truth was cheering. What these Sociétés de l'Harmonie" were, may be gathered from the following details. They seem to have been agreeable enough, and were founded probably on the classic model, of the rites of Venus and of Pan.

"All Mesmer's arrangements were well adapted for producing a powerful impression on the minds of his patients. They were all assembled in one large apartment, and seated around an impressive-looking apparatus, while a dim mysterious twilight only was shed upon them from without, or from a few lamps; notes of soft music were heard from a concealed corner; and young men, selected for their beauty and robust forms, began to perform the various manipulations of the art, passing their hands over the face, bosom, body, and limbs, of each of those who sat around, in silent expectation of some extraordinary sensation, of which they had heard from Mesmer or their friends. Is it wonderful that ardent and voluptuous French girls should blush, or that their pulses should quicken, or their breathing become rapid, under such excitement as this? or that a worn-out old *debauché* should perceive unusual sensations? or that the nervous and debilitated should tremble at such treatment? When all that excitement of the animal passions—imagination, anxiety, love, and lust—could accomplish, then Mesmer himself entered the apartment, habited in delicately-coloured silk, and wearing an air of deep gravity and importance: walking about as if endowed with magic power, he presented to each in turn a rod, or his hand, which *they were told and believed*, was charged with the magnetic virtue. Some of them were then at once seized with hysterical fits, and all the others, by imitation, followed them. 'As soon as one began,' says the first report, 'another succeeded.' 'The convulsive state,' says M. Du Potet, 'once excited in a patient, never failed to manifest itself in all the others.'

Here, then, we have every circumstance pointing out the true nature of the crisis. The patients were nearly all women, and these the most frivolous of the voluptuous court of Louis XVI., during the most licentious period of modern France; there was therefore the favourable predisposition in ardent animal passions, in a superficially cultivated intellect, in a body weakened by dissipation, and a mind yearning for some new and powerful excitement; there were expectation, anxiety, and perhaps some fear of strange and novel feelings—for Mesmer never failed to let his patients know what might (and *therefore would*) be the effect of the magnetic influence: there were the awe with which ignorance looks up to one supposed to possess supernatural power, the heated passion excited by the meeting and the contact in no decent degree of the opposite sexes, and perhaps some modest fear."*

The world must feel a lively curiosity to learn the fate of a man like Mesmer—a man destined to revolutionise science, and so admirably qualified to improve our moral natures. For the benefit of his health, he withdrew to Spa, whither he was followed by numerous persons of rank and fortune, who were still desirous of placing themselves under his treatment. His personal history at this epoch is not detailed by M. Dupotet. Perhaps we have lost much by our unacquaintance with it. But we are delighted to add, that, after receiving a subscription, which was raised in compensation for his having devoted his life to the promulgation of so important a discovery, he retired to his native place, where he died, much beloved and honoured, on the 5th of March, 1815, at the advanced age of eighty-one. It is not always that the real benefactors of their species are so substantially rewarded. Mesmer, St. John Long, Morison, shall we say Hahneman, may be pitted against Galileo and Harvey. The appreciation of the former may serve, perhaps, to compensate for the wrongs of the latter.

---

* The preceding passage is from the second of a series of letters on animal magnetism in the Medical Gazette. The writer is not imbued with that "aptitude à croire" which is so proper in the examination of new and incomprehensible doctrines. On the contrary, he displays a lamentable disposition to disbelieve what seems impossible, to laugh at what appears ridiculous, and explain strange facts on natural rather than miraculous grounds.

The Revolution shook the science of Mesmerism, and disturbed the masonic reveries of the Sociétés de l'Harmonie. But when the political storm began to break, Mesmerism reared its head in Germany and France, and participated in the advances of the other divisions of human knowledge. Academies *franc'd* it—governments legalised it—fools laughed at it—and choice spirits embraced it. Among these latter, none were more choice than the Marquis de Puységur, and the Abbé Faria, gentlemen who, from their professions, must have been qualified to reason well upon medical matters. The Marquis was the discoverer of magnetic somnambulism. The Abbé opened in Paris an institution for magnetism which attracted the attention of many scientific men. He was himself endued with the magnetic power to a degree which would have appeared incredible, were it not a notorious fact that its intensity augments with exercise. The daily increasing converts to magnetism attached themselves to this man of wonders ; they listened with interest to his lectures, and learned by his instructions how themselves to conduct the operation.

A science, like this, could not long remain, like the French Republic, " one and indivisible." Dissent became inevitable.

"Three separate schools of magnetism arose:—1st, the original school of Mesmer. This prevailed principally in Paris. Its doctrines were very similar to those of the Epicurean philosophy, as described in the poem of Lucretius. Its disciples believed in the existence of the universal fluid above described, and conducted the operation physically,—that is, by passing the hands immediately over, or at a short distance from, the body of the patient. 2nd, the school of the Chevalier de Barbarin. This was founded at Lyons, and, although it had many partisans in France, prevailed principally in Sweden and Germany. Its principles remind us of the Platonic philosophy; its disciples maintained that the magnetic operation depended entirely upon a pure 'effort of the soul,' and was to be conducted only upon psychical principles. They were therefore termed spiritualists. Lastly, the school of the Marquis de Puységur, founded at Strasburg, the disciples of which, professing to be guided only by observation, called themselves experimentalists. The characteristic feature of this school is, that it combines the physical treatment of the school of Mesmer with the psychical treatment of that of Barbarin." 18.

It cannot be otherwise than creditable to the general stock of common sense in France, that the belief in animal magnetism spread with rapidity. Shoals of young women were brought " en rapport," and not a few old ones were converted. Members of the Chamber of Deputies opened their saloons, the proselytes grew clamorous, the public expectant, and the Academy was urged to reconsider its ancient verdict. One of the members proposed that a new special commission should be appointed, for the reconsideration of the subject. It is instructive to observe the contest of ancient prejudice and infant truth—of the old bigotry and the young enthusiasm of the Academy.

" This," says M. Dupotet, " was a perplexing proposition; the Academy knew not which side to take. It was composed of many members who were convinced of the truth of animal magnetism, and many who were undecided, and desirous of receiving further information. The older members were unwilling that the magnetisers should derive any importance from their decision; they were even apprehensive that the bare nomination of a committee of inquiry would lead to an impression that they were favourably disposed towards the doctrine; and several were naturally enough disinclined to disturb, much less to rescind, the decision against it contained in the old report. But the case was urgent; they therefore adopted an intermediate course. They appointed a committee, MM. Adelon, Burdin, Marc, Pariset, and Husson, to make a preliminary investigation, and report whether the Academy, without compromising itself, could appoint a new commission. This was surely proceeding with all due precaution,—and what was the result? The committee convinced itself that the evidence was sufficiently strong in favour of animal magnetism to warrant the Academy in authorising another official investigation; and on the 13th of December, 1825, some months after their appointment, the commissioners, faithful to their duty, presented themselves to deliver their report. There was on that day an excitement among the members in the council-room which did not terminate with the sitting. Three days were occupied by the debate, when at length the Academy decided, by a majority of ten, in favour of the appointment of new commission. Eleven members were therefore nominated—viz., Bourdois de la Motte (the President), Fouquier, Gueneau de Mussy, Guersent, Husson, Itard, Marc, J. J. Leroux, Thillay, Double, and Magendie." 23.

For five years the Commission were silent. Perhaps they were buried in a magnetic slumber. In this state of somnambulism, they saw, they felt, and were convinced. At the end of the five years devoted to the examination of this wonderful science, they announced that their report was ready, and, on the 20th of June, it was read by M. Husson at the bar of the Academy. The whole of the Report could not be read on the first day, and its conclusion was deferred until the next meeting. Let M. Dupotet describe the scene.

" This was the day of the grand battle;—I say, battle, for on this occasion there was a general affray among the members of the Academy. While the report was being read, they listened with uneasiness to the facts detailed; but when the higher phenomena of lucidity were described, a general murmur, not very flattering to the commissioners, prevailed, which gradually increased, until several Academicians started from their seats, and apostrophised, in unmeasured terms of indignation and contumely, the men who had conscientiously related the facts which they had seen and attested. An outcry was raised on all sides against the commissioners; but, without being disconcerted, many members of the Academy, who believed in magnetism because they had themselves examined it, vindicated them, and retorted:—' You do not believe in magnetism—be it so; but in this very place the circulation of the blood was denied; yet the blood does circulate! Here they who practised inoculation were denounced as impostors, and the inoculated as dupes and idiots; yet was inoculation no imposition or idiotcy;—here, also, the physicians who first employed tartar emetic were put on their trial, and expelled the Academy; yet we have now for colleagues men who employ it in enormous doses!' Another exclaimed, with stentorian voice, ' This Institution ridiculed those who affirmed they had seen stones fall from the sky; yet meteoric stones do fall!' Thus the sanctuary of science was on a sudden converted into an arena of Babel-like confusion, because a few of the learned members of the Academy were not prepared to accredit the facts which the commissioners, whom they had themselves delegated with the inquiry, reported to them. But it was necessary to terminate so turbulent a discussion; and then came the question whether the report should be published or not, which, after another stormy contention, was decided in the negative." 26.

We blush for the Academy. Its Commissioners had been magnetised and the witnesses of magnetism for full five years—they had seen phenomena which no one can believe without seeing, and which some will not believe when they do see them—they had yielded their assent to the greatest apparent absurdities, and consented to seem to verify the adage, that, "doubtless the pleasure is as great of being cheated, as to cheat"—and the reward they received from the whole Academy was the rejection of their elaborate report —a rejection which virtually said, "Gentlemen of the Commission, though you have been at the pains to write yourselves down asses, we of the Academy don't intend to follow your example." The denunciation of antimony was not half so bad as this, and a Piron of these days would re-write his epitaph :—

Ci git Piron, qui ne fut rien ;
Pas même Academicien.

But the folly of the Academy was not at its height. Not content with thus stultifying itself in its old commission, it has subsequently appointed a second. M. Dupotet charitably avoids a full account of the operations of this second batch of Commissioners. The first decided in favour of animal magnetism. As a lover of his science and convinced of its truth, M. Dupotet would have been infamous had he not dwelt on what was calculated to support it. But the second academical commission unfortunately has arrived at a counter conclusion, and has done Mesmerism the injustice of making it look like humbug. Surely it was not incumbent upon M. Dupotet to draw attention to this. He therefore concludes his history of magnetism with the favourable report of the first commission, and an offer to convince the whole world upon terms of the most moderate description.

We are almost tempted to imitate the plan of M. Dupotet, and to dismiss in silence the scandalous report of the second commission. But two considerations have determined us to insert it; because the triumph of magnetism will be the greater, the stronger the apparent facts opposed to it: and because, it is only necessary to expose the ungentlemanly precautions of the Reporters against imposition or error, to induce all properly-constituted minds to form a decided opinion on the subject.

It seems, then, that the Commission was composed of MM. Bouillaud, Cloquet, Caventou, Cornac, Dubois (of Amiens), Emery, Oudet, Pelletier, and Roux. M. Roux was chosen President, and M. Dubois, Secretary and Reporter.

The whole of the Commission, except M. Oudet, met at the house of M. Roux, at 7 P. M. of the 3rd of March. At a quarter to 8, M. Berna introduced a young girl of 17 or 18, of a constitution apparently nervous and delicate, but with an air sufficiently unconcerned and resolute.

"The programme of the evening's experiments, which we had sent to M. B., presented eight experiments. The following are their titles, literally copied, for the language does not belong to your Commissioners.

1st, Somnambulism.

2d, Proof (constatation) of insensibility to pricking and tickling.

3d, Restitution, by the mental will, of the sensibility.

4th, Obedience to the mental order to lose motion.

5th, Obedience to the mental order, to cease answering in the midst of a conversation; and to the mental order, to answer again.

6th, Repetition of the same experiment, the magnetizer being separated from the somnambulist by a door.

7th, Waking.

8th, According to the mental order which shall be enjoined in the somnambulic state, persistence in the restoration of the sensibility, and also persistence of the power of losing or recovering this sensibility at the will of the magnetizer.

The young girl introduced to your Commissioners was received with caution and affability: we conversed with her on indifferent things, and then, to determine, before any attempt at magnetization, how far she was in her ordinary state sensible to pricking, needles of moderate size, brought by M. Berna himself, were stuck in to the depth of about half a line. Their points were made to penetrate into the hands and neck of this young person, and then, when asked by some of the Commissioners, with an air of doubt, if she felt the pricking, she answered positively to M. Roux and M. Caventou, that she felt nothing; her figure, moreover, did not express any pain. Let us remind the Academy, that she was at present perfectly and normally awake, by the confession even of her magnetizer, who had not yet commenced any of his manœuvres. This scarcely agreed with the programme, for the insensibility ought not to have been acquired till in the state of somnambulism, or after and by the mental injunction of the magnetizer,—an injunction which could not be given except in this state.

Your Commissioners were a little surprised at this singular commencement. What! do you feel nothing? they said to her. But are you absolutely insensible?—Then she finished by confessing that she felt a very little pain.

These preliminaries completed, M. Berna made her sit close by him. *Tête à tête* with her, he appeared at first to contemplate her in silence, without practising any of the movements called passes; after a minute or two, he said to your Commissioners, that the subject was in somnambulism.

The girl's eyes were covered with cotton and a bandage.

M. Berna had no other proofs to give your Commissioners of the pretended state of somnambulism, which, besides, he did not define theoretically, than the experiments comprised in his programme. Then, having again contemplated his somnambule at a very slight distance, he announced that she was struck with general insensibility.

What now could be the part your Commissioners should perform? Physicians, surgeons, natural philosophers, all knew that the proofs of the abolition of sensibility are of two kinds; that the one are deduced from the assertions of the subjects—the others from the signs of the external deportment—the language of action. Now the first might be considered as null, when concerned with individuals whose interest is to deceive and lead into error. The mute signs drawn out by pain remained; but then, on the one hand, the intensity of the pain, and on the other the firmness of the patients, had to be taken into consideration. In the present case the intensity of the pain was not to pass certain limits rigorously fixed by M. Berna.

However, some of your Commissioners, armed with needles, among others MM. Bouillaud, Emery, and Dubois, set them to prick the poor girl. By word she complained of no pain: her features, as far as we could judge, expressed no painful sensation;—we say as far as we could judge, for her eyes being covered with a large bandage, half her features were concealed from us,—we had scarcely any thing left to observe but the forehead, the mouth, and the chin.

M. Bouillaud, in his trials, did not go beyond the agreed limits; but the reporter, having stuck the point of his needle under the chin with more force, the somnambule made at the moment, and with vivacity, a movement of deglutition. M. Berna perceived it, and gave new cautions.

Touched with the end of the finger by M. Cloquet in the surface of the hand, the somnambule said she felt this impression; so that independently of the perception of temperatures, she had still preserved that of touches,—which, in the system of M. Berna, would add new restrictions to this pretended general loss of sensibility. However, the magnetizer, pursuing the course of his experiments, told the Commissioners that he was going, by the sole and tacit intervention of his will, to paralyse, either from sensibility or motion, any part of the girl's body that they would wish. The following conditions were then made:—

That M. Berna should maintain the most perfect silence, and should receive from the hands of the Commissioners, papers, on which should be written the parts to be deprived of or endowed with either sensibility or motion; and that he should let them know by closing one of his eyes that it had been done, and that they might verify it. He said he could not accept these conditions, and gave for reason, that the parts pointed out by the Commissioners were too limited, and that besides all this was out of his programme, and he did not understand thus the precautions that would be taken against him.

Your Commissioners had written—1st, to deprive the chin of sensibility; 2d, the right thumb; 3d, the region of the left deltoid; 4th, that of the right patella. M. Berna had written in his programme, that to show us the sufficiency of his action, he would raise his hand towards us, and that this should be the sign in this experiment as in all the others. This was one of the precautions he had planned; but as your Commissioners took good care to look to all these points, they thought they might require of M. Berna, that instead of raising his hand for a signal, he should be content with closing one of his eyes.

As to limits, M. Berna had pointed them out in his programme. For sensibility—1st, the whole of the body; 2d, a part of the body only. For motion, he had written—*a*, the two arms; *b*, the two legs; *c*, an arm and a leg; *d*, a particular arm and leg; *e*, the neck on the right or left side; *f*, the tongue. But here we must explain to the Academy what M. Berna understood by paralysis, and by the verification of this paralysis.

All the evidence the Commissioners were allowed to have of its existence, was, that when told to raise her arm, &c., the somnambule did raise it or not; in the latter case—that is, if when told to do it, she did not raise her limbs or move her head, or talk—she was to be considered as paralysed by the tacit will of M. Berna, and that all this depended on the agency of animal magnetism. Besides this, the Commissioners were to make haste with their observations. If the first trials did not succeed, they were to be repeated till paralysis was produced—very good plans for the public, but such as men of science, who were to give an account of their commission, could not exactly comply with. M. Berna then said he would do no more at this meeting, but would wake the somnambule, and at the same time restore her sensibility. M. Bouillaud, at his invitation, was first to place himself behind the girl, ready to prick the back of her neck when the magnetizer gave him the signal. He, M. Berna, placed himself opposite the girl in the same position as the first time. Wake! said he, twice. Then he raised the bandage and the cotton from her eyes, leaned towards her again, put his left arm behind her, and stopped M. Bouillaud, who was of course going to prick her too soon; then leaning towards the girl again, whose eyes were perfectly open, he looks at M. Bouillaud; that Commissioner then pricked the somnambule, who turned her head aside, and M. Berna cried out—There, the sensibility restored! Your Commissioners make no reflection on the value of the facts which M. Berna had shewn them."*

It must, we think, be admitted that the Commission was composed of very unreasonable persons. They ought not to have pricked the young lady before she was duly magnetised. Their doing so spoilt the experiment. Her insensibility, when magnetised, would have been a thousand times more satisfactory, if these officious Commissioners had not discovered her insensibility before she was magnetised at all.

The Commissioners seem puzzled for means of determining the reality of the magnetic somnambulism. But who, we would ask, can be better judges of it, than the magnetisers who induce, and the patients who feel it? They must know what somnambulism is, much better than a host of mere physicians, surgeons, and natural philosophers. If magnetiser and patient then concur in the somnambulism of the latter, it is exceedingly uncivil, not to say absurd, in any one to doubt it. The superiority of assertion to all ordinary evidence is clear, indeed, in the present case. For, when the Commissioners attempted to corroborate M. Berna's declaration, by sticking pins into the somnambule, either she displayed no more insensibility than she had done before the induction of somnambulism, or, when an unlucky pin was poked too far, a vivacious movement of deglutition occurred at the most inopportune moment. If M. Cloquet, too, had refrained from touching the patient's hand with his finger, we should not have been annoyed with the perplexing fact of the somnambule's having said that she felt it.

M. Berna was perfectly right in refusing to do what was out of his programme. There is a regular way of transacting business in animal magnetism, as in other concerns; and a person who had been accustomed to move her arms and toes in a certain order, had no right to expect that the routine should be interrupted. We are all aware of the force of habit. The somnambule had probably performed her part on numerous occasions. If let alone, everything would have gone on satisfactorily. Her legs and arms would have gone up or down at the proper time and place, and she would have balanced herself on her nose or her rump, in a fashion that would make a Mahometan dervise die of pure envy, and would have gone very far towards convincing half the old women of Paris, in petticoats or out of them.

M. Berna did well to bring the exhibition to a close. We will now detail from the same report the proceedings of a second sitting.

"At half-past eight in the evening the same somnambule and all the Commissioners being assembled, and the somnambulism having been produced, M.

---

* This Translation of the Report of the Commission is copied from the Medical Gazette. It is impossible to curtail it with advantage.

Bouillaud requested in writing that M. Berna would have the goodness to paralyse the right arm only of the girl, and when it was done to indicate it to him by closing his eyes. M. Berna, then sitting near the girl, lowered his head towards her hands, which she held in her lap. The reporter, led by what M. Berna had said, viz. that there should be no contact either immediate or mediate between him and the somnambule, interposed a sheet of paper between his face and her hands.

Presently M. B. made the agreed sign, that his silent will had been sufficiently powerful to paralyse the right arm only of his somnambule. M. Bouillaud proceeded to verify the fact, and for this purpose asked the girl to move successively this or that limb ; when he came to the right leg, by way of elimination, as one may say, she answered that she could not move either the right leg or the right arm.

Remember that M. Berna's programme stated that he had the power of paralysing either a single limb, or two limbs at once ; we chose a single limb, and there resulted by his own confession, spite of his will, what he called a paralysis of two limbs. The experiment missed, and it was necessary to pass to another ; for we had not the politeness, notwithstanding the terms of the programme, to re-commence till it succeeded, which certainly must have been soon, since we had only to choose between four limbs and the tongue." 920.

It must be confessed that the Commission reason plausibly, and, under ordinary circumstances, their decision would be laudable. But, perhaps, the common rules of evidence should be dispensed with in so uncommon a case as that of magnetism. As that is opposed to common-sense, it seems hardly fair to try it by common-sense procedures. This is making the defendant judge. For our own parts, we are convinced that no man of common sense ought to trouble himself much about Mesmerism, and few, we are pleased to notice, do. The science is of too high an order for individuals possessed of this homely character of mind.

If " half a loaf is better than no bread," it seems to us that à fortiori two loaves are better than one. We are almost tempted to assert that Mesmerism is something plus the truth. When the magnetiser signified by a wink that he had paralysed the girl's right arm, and it turned out that her right leg was paralysed too, surely that was the strongest evidence of the powers of animal magnetism. It did twice as much as was expected. Can that be said of many sciences ? Let us pass to the third sitting.

" On the 13th of March, at half-past seven in the evening, another meeting was held, and the same proceedings were gone through. ' Remove from your somnambule,' wrote M. Bouillaud on a piece of paper, ' remove the power of hearing me, while you stand behind M. Dubois, and then, touching his shoulder, let me know that it is done.'

The magnetiser agreed, but wished that the somnambule should be placed very near M. Dubois, who was to act as a screen, and that she should be a foot off him. This was punctually done ; the reporter (M. Dubois) made M. Berna go behind him, and hid from him, at least in part, the somnambule, while M. Bouillaud conversed with her in the situation just mentioned ; but long before the magnetiser had made the agreed sign, she seemed no longer to hear M. Bouillaud, which shewed that the magnetizer's will had acted quicker than he thought ; but when the signal was given, then she begins to answer M. Bouillaud, which was precisely the contrary of what ought to have happened."

" But as the magnetizer had from the first moment of his transactions with us spoken of these marvellous facts of vision without the assistance of eyes, and of those famous transpositions of the senses, so much talked of in the archives of animal magnetism, you may imagine how desirous we were of seeing such experiments; never had any thing like it been tried before an academic commission." 920.

Never had such an issue been tried before any commission, save such as the audience of some peripatetic Katerfelto may be deemed. The results were worthy of the actors and the science.

"On the 3d, your Commissioners met again, and witnessed the following facts:—At eight in the evening we met at M. Berna's. He was placed by the side of a woman aged about 30. After our arrival, he covered her eyes with a band, and then told us that she was in a state of somnambulism, and began to talk aloud with her.

Interrogated by her magnetizer (for none of us spoke at this meeting)—interrogated if she saw what was passing around her, this woman declared, that to distinguish objects better, she must turn so as to face him. M. Berna approached her, so that their legs touched, notwithstanding what was said in the programme; but still a blow, this was secondary for facts of vision, without the assistance of eyes.

Your Commissioners, attentive to what was passing, were, however, penetrated with this idea, that in this sitting there would be two kinds of facts—1st, Those whose solution was proposed to the woman said to be in somnambulism, but which were known to M. Berna. 2d, Facts whose solution was also proposed to her, but which were unknown to M. Berna, and which would be in part arranged without his knowledge. The latter would have a great value, an absolute value, independent of localities and the morality of the actors, and ought to carry conviction with them. The others would remain subject to various interpretations—to objections more or less founded, and therefore might leave doubt in the mind. Thus, to cite a first instance, the magnetizer commenced by asking the woman how many persons there were present? Several gentlemen, she answered; at least five. This first fact was as well known to M. Berna as to us; and we may add that, approximatively, she herself might know it, since her eyes were not covered till after our arrival.

At the invitation of the magnetizer, who directed every thing in this solemn sitting, the reporter was to write on a card one or several words, that the somnambule might read them. The Commissioners, thanks to the officious care of M. Berna, had at their disposal, on a table, two packs of cards, one perfectly plain, the others playing cards. Thus you see the order of the sitting had been obligingly regulated by the magnetizer; there were no more of those hesitations and those uncertainties which had in some measure disturbed the other sittings; here every thing was arranged beforehand." 921.

Nothing could be more civil on M. Berna's part, nor, indeed, could any thing be more judicious. It is obvious how much more satisfactory it must be for things to run in a groove. When things turn up the reverse of what was looked for, men's minds are unsettled, and no beneficial conclusions can be formed. If, for example, M. Berna's patient had been blind-folded before the Commission entered, it is possible that she might have blundered in reference to their numbers. Her stupidity would have prejudiced magnetism. M. Berna would have been inexcusable to have omitted any precautions against so injurious an accident. But to proceed.

"However, the reporter wrote on a blank card the word PANTAGRUEL, in printed and perfectly distinct letters; then placing himself behind the somnambule, he presented the card close to her occiput. The magnetizer, seated opposite M. Dubois—that is, in front of the woman—could not see the characters traced on the card; it was a fact of the second order, mentioned above, that is, decisive in itself.

The somnambule, interrogated only by her magnetizer as to what was put behind her head, answered, after some hesitation, that it was something white—something resembling a card—a visiting card. Hitherto, as you may believe, there was nothing to surprise us. M. Berna had said aloud to the reporter to take a card, and write something on it. The somnambule might therefore say she saw something white, like a card; but as soon as she was asked if she could distinguish what there was on this card—'Yes,' answered she firmly, 'there is writing on it:' an answer which again did not surprise us. 'Is it small or large, this writing?' 'Pretty large,' she replied. Here, as you see, the serious difficulties commenced, and the somnambule resorted to approximations. 'What is written on it?' continued the magnetizer. 'Wait, I cannot see plain. Ah! there is first—an M,—yes, 'tis a word beginning with an M.' Such were the first answers of the somnambule.

M. Cornac, unknown to the magnetizer, who alone put questions to the somnambule, then passed a perfectly blank card to M. Dubois, who immediately, and unknown to M. Berna, substituted it for the one which had the word Pantagruel on it. The somnambule still persisted in saying that she saw a word beginning with M.—M. Berna, who did not suspect in any way our contrivance, still pressed her with questions; she was invariable; she could only, she said, distinguish a single letter, an M. At last, after some efforts, she added, with some doubt, that she saw two lines of writing.

MM. Oudet and Cornac were then placed behind her; she said she could see one of these gentlemen, M. Cornac. She was asked if he was large? Not very, said she—not so large as you. She was speaking to M. Berna, who alone conversed with her.

M. Cornac, with the consent of the magnetizer, presented in his turn a card to the occiput of the subject, on which he had written the word *Aimé*. She distinguished, she said, some writing, but could not say what it was, what it signified. M. Cornac drew a long purse from his pocket. It is something round, she said: then putting his purse in his pocket again, he presented his hand alone. She said she still saw something round." 921.

We confess we feel a difficulty in explaining these circumstances. We cannot understand why this young woman could not read with her occiput. That animal magnetism would enable her to read with her toe-nail, no candid nor reasonable man can doubt. The thing is itself a charming as well as a likely fact, and besides the magnetizers and the magnetized both say that it is so. They must know best, that is certain. Away with the cold scepticism that answers "impossible." The word is not only not French, it is of no language in these glorious days of homœopathy and Mesmerism.

It would, no doubt, have tended to prevent the occurrence of these distressing mistakes, if M. Berna had been made acquainted before-hand, with all that was proposed to be done. We would suggest to all future Commissions to act in a free and gentlemanly manner, and afford the magnetizer and the patient every possible facility. Let us not witness the Vandalism of men of learning throwing obstacles in the way of science.

"After these first attempts, the somnambule complained of being dazzled; that she was annoyed by light. Yes, answered the magnetizer, by fogs, wait—and by means of some fresh passes, he said he had relieved her." 921.

How wonderful a thing magnetism is. Here was a young woman, who had been for some time blind-folded, reading wrong with her occiput, and when she could not see such an object as a purse, she complained that she had all along been dazzled with the light. This, no doubt, was the cause of her blindness. But the curious thing is that, after all, she was not dazzled by light, but fogs. M. Berna, at all events, said so, and M. Berna must have known. Having driven away these dazzling fogs, by his passes, the patient was prepared by M. Berna for any thing. We shall see how she got on.

" The reporter charged with taking notes, was writing at this moment within two steps of the somnambule: the point of the pen was heard running along the paper; the somnambule turned aside and raised her head, as if endeavouring to see under the lower edge of her bandage. The magnetizer quickly asked if she saw that gentleman. Yes, she said, he is holding something white and long (the reporter was writing on a paper longer than broad.) He then approached the somnambule, placed himself behind her, and ceasing to write, put his pen in his mouth. M. Berna then interrogated his subject in the same manner, that is on facts of which he had knowledge as well as we. Do you still see, he said, that gentleman behind you? Yes, said she. Do you see his mouth? Not very well. Why? There is something white and long across it. The magnetiser cast a look of satisfaction towards us, and recommended the reporter to make special note of this fact

We have taken care not to forget it; but what is its value or importance in relation to the doctrine of animal magnetism? On the one hand, the somnambule knew that she had turned towards some one writing; the distinct noise of the pen on the paper was enough to make this certain, even admitting that she had not seen under her bandage, a trial which she made without opposition on our part; because, as we have already said, we wished to let the magnetizer act without the least appearance of constraint. The reporter still writing, placed himself behind the woman, and then only ceased to write, and put his pen between his teeth. The magnetizer did not take another commissioner for the subject of his questions; but addressed to the somnambule, assuredly without wishing it, a question too indicative—too particular. Do you see that gentleman? Well: but why say—Do you see his mouth? What is there in his mouth? the somnambule might at once ask herself. He has been writing—he has placed himself behind me while writing—can it be his pen that he has put in his mouth?—it is something white and long.

These reflections came at once into our minds, and removed from this fact the value which it might perhaps have had without these circumstances." 922.

The Commission betray throughout these observations, that desire to discover easy and natural explanations for seemingly mysterious facts, so contrary to true science. Why elaborately account for the patient's declaring that the Reporter had something white in his mouth, when the fact that she saw it all with her occiput is so evident?

" In the facts which are about to be presented to you, things could not go on in this manner; varied interpretations were not possible: let us see what was the result.

On a fresh invitation of the magnetizer, M. Dubois wrote in large letters on a card of the same size as the first, a single word—MISERE, without letting the magnetiser know what it was, and presented it for the somnambule to make out, placed as usual at her occiput. M. Berna's request had been made aloud; the somnambule did not fail to say, without hesitation, that she saw a card, and that there was writing on it. Solicited as before, she seemed to make efforts to distinguish the letters; at last, after great hesitation, she said the word began with a T. The reporter substituted a blank card, and presented it; but neither the somnambule nor the magnetizer could in any way perceive the substitution. Interrogated as to the number of letters, she said she saw five or four. We have said the card was perfectly blank.

Now, gentlemen, we are coming to facts more decisive, more curious, and in which the lucidity of the somnambule was to appear in full evidence. We have already said that M. Berna had prepared on one of the tables in his apartment a pack of playing cards. This time, again addressing the reporter, he asked him aloud, and without leaving his intimate relation with the somnambule, to take a playing card, and place it at her occiput. Is it to be a court card? asked the reporter. As you please, answered M. Berna.

This perfectly natural question the reporter had made at first without reflection, quite innocently; but as he went towards the table on which the pack of playing cards had been previously laid out, the idea struck him not to take either a court or a common card, but while pretending to take a playing card, to take instead, a perfectly blank one of the same size, still unknown to M. Berna, and we need not add, to the somnambule, since she could not perceive substitutions made an inch from her occiput, to which her vision had been transposed.

Then, with his blank card, the reporter placed himself at her occiput, and held it behind her. The magnetizer, seated before her, magnetized with all his force. The somnambule is interrogated,—hesitated,—made efforts, and said she saw a card; but the magnetizer was not, any more than we, contented with so little. He asked her what she remarked on the card? She hesitated, and then said there was black and red!

The Commission let M. Berna continue his manœuvres and his solicitations, that he might clear what still appeared very confused, before the woman's transposed sense, and which as yet consisted only of a little black and red. After some fruitless essays, the magnetizer, undoubtedly but ill satisfied with the functions of the transposed visual sense, invites the reporter to pass his card before the head of the somnambule, close to the band covering her eyes: this was, it may be said, changing the terms of the question, and even of the magnetic doctrine; it was giving up the transposition of the senses, to substitute *clairvoyance* through a bandage. But it mattered little: the reporter passed the card as the magnetizer wished, but he took care to pass it quickly, and so that M. Berna might suppose he saw only the naturally white back of the card, while the coloured part was turned towards the somnambule's bandage.

The card once in this new position, the magnetiser continued his manœuvres, and solicited the somnambule. She confessed that she saw the card better; then added, hesitating, that she saw a figure. New urging from M. Berna,—new solicitations! The somnambule, on her part, seemed making great efforts. After some trials, she declared plainly that she saw a knave!! But this was not all: it remained to say what knave, for there are four. Proceeding, without doubt, by way of elimination, she answered her magnetizer that there was black by the side of the knave. Still this was not all: there are two knaves with black at their sides. New urging by the magnetizer,—new efforts by the somnambule,—new and profound attention by the Commissioners. At last she has it.—It is the knave of clubs!

M. Berna, thinking the experiment finished, took the card from the reporter's hands, and in presence of all the Commissioners, sees and assures himself that it is entirely blank." 923.

This is unaccountable. As M. Berna was before the girl, we cannot help thinking that she was right in protesting that she saw a knave. There was clairvoyance, we conceive, in that, and, when properly considered, strong evidence of the truth of Mesmerism.

" As a last operation, leaving both the writing and the playing cards, M. Berna asked M. Cornac for an object he had brought with him, adding, that he would present it in his closed hand before the somnambule's bandage. This object, which we do not mention the name of yet, was given by M. Cornac to M. Berna, and he, with one hand, presented it close to the somnambule's bandage, and with the other endeavoured to act magnetically on her, and then recommenced the inquiries, solicitations, urgings, &c. She, who had not lost courage, appeared to make great exertions. Her magnetizer asked her if she could distinguish what he had in his hand. Wait! said she. Then, after these feigned or real uncertainties, she said it was something round: then, still pressed with questions, she added, that it was flesh-coloured,—that it was yellow,—and lastly, that it was of the colour of gold. At new and incessant questions, she added, that it was about as thick as an onion,—that it was yellow on one side, white on the other, and that, lastly, there was black above it.

Here she complained, and wished, she said, that her magnetizer would finish and wake her: she urgently asked it. Not yet, answered M. Berna,—when you have answered my questions; and then he agitated his hands before her, to drive away obscurities and fogs. Pressed anew to tell the name of the object presented to her, she repeated that it was yellow and white. Do you say it was white? asked M. Berna. (Here the Commission incidently remarked, that M. Berna was perhaps wrong in recalling only the word white: there was in this, as you will presently see, something too indicative—too special.) But the somnambule said positively yellow on one side, white on the other, with black above.

Have you, said M. Berna, such an object? No, said she. Have I? Ah! yes, you have that. But, rejoined he, if you had it, what would you do with it? I would put it on my neck, Solicited, for the last time, to explain herself better, —to say at least the use of the object, if she could not tell the name,—she seemed to collect all her powers, and then uttered only the word *hour;* then at last, as if suddenly illuminated, she cried out, it was to see the hour. M. Berna returned M. Cornac the mysterious object: it was a silver medal, of the weight and size of a piece worth three francs; on one of its surfaces there was a caduceus, on the other two capital letters." 923.

We will do M. Berna the justice to say that he did his best throughout, to acquaint his patient with what was going on, and, no doubt, had fortune been on his side, he had drilled her so scientifically, that all would have gone well. This laudable love of truth and science was not so successful as could have been desired.

Perhaps it is not necessary to subjoin the conclusions at which the Commission arrived, and which it specified in the Report to the Academy. The experiments speak for themselves. But we are tempted to quote some of the observations of the Commission, because they communicate particulars imperfectly understood from the details of the experiments only.

*a.* "According to the terms of the programme, the second experiment was to consist in the proof of the insensibility of the subjects. But after having mentioned the restrictions imposed on your Commission—that the face was put out of sight and removed from every trial of this kind—that it was the same for all the parts naturally covered, so that there remained only the hands and neck;—after having reminded you that on these parts we were permitted to exercise neither pinching nor scratching, nor the contact of any body, either on fire or of a slightly raised temperature, but were limited to the sticking in of needles to half a line deep;—after recalling all these restrictions, we are justified in deducing from these facts—1st, that none but very slightly painful sensations could be excited; 2d, that these could be produced only on parts perhaps habituated to this kind of impression; 3d, that this kind of impression was always the same, and that it resulted from a kind of *tatonage;* 4th, that the features, and especially the eyes, where painful impressions are more especially indicated, were hidden; 5th, that in consequence of these circumstances, an *impassibilité,* even complete and absolute, could not be a conclusive proof to us of the abolition of sensation in the subject in question." 955.

The objections to the proofs of insensibility adduced by the magnetizers, are specious and plausible. But, since it is certain that the patients *are* insensible, it would be cruel to burn and torture them for nothing. The magnetizers, then, do right, to prescribe such gentle injuries as habit, independently of magnetism, could render tolerable, and such as can do no real mischief.*

*b.* The Commission dwells at length on the absence of satisfactory proof of insensibility and paralysis on the part of the magnetised. The assertions of the parties concerned are the main, indeed the only evidence. But those parties must know best, and it is not handsome to suspect them of delusion or falsehood. If appearances are against them, as in M. Berna's experiments, appearances are proverbially deceitful; and, though they do not seem so, M. Berna may still be a sensible and candid man, and his patients very honest and decent young women. In weighing evidence we ought to lean to the side of implicit faith. It is to be lamented that the Commission has done just the reverse. Being the actual spectators of the circumstances, truth would naturally make a strong immediate impression on the commissioners, and much importance would, of course, have been attributed to an expression of conviction from such a quarter. But as that conviction has been counter to their claims, the magnetizers have acted properly in attaching no importance to it. How could they feel any thing but pity and contempt for such conclusions as the following?

"Despairing of proving the transposition of the sense of sight—the utility and superfluity of the eyes in the magnetic state, the magnetizer wished at least to take refuge in the fact of *clairvoyance*, or vision through opaque bodies.

You know the experiments made on this subject; they present the capital conclusion, that a man placed before a woman cannot give her the power of seeing through a bandage. But here a more serious reflection presents itself: admitting for a moment the hypothesis (which is very convenient for the magnetizers), that in many circumstances the best somnambules lose their lucidity, and, like common mortals, can no longer see with the occiput or the stomach, or even through a bandage, what are we to conclude of this woman from the minute description which she gave of other objects than those presented to her? who described a knave of clubs on a perfectly blank card—who, in an Academic medal, saw a gold watch, with a white face and black letters—and who, if urged, would perhaps have finished by telling the hour that this watch marked?" 956.

We had hoped, for the honour of animal magnetism, that the Commission was composed of men opposed, *à priori*, to the science—of men of the stamp of the bigots, who, alas! form the bulk of the profession. Had that been so, the uncivil experiments, and the unfavourable conclusions would have been at once explained. Unfortunately it is not so. The Commission itself, sensible, we hope, of the decided impression that its report would cause, takes care to rebut the accusation.

"Arriving at our own Commission, we must first remind you that you had

---

* The capability of enduring pain conferred by the stimulus of imposture or enthusiasm, may be easily seen in our army or navy, in the dervishes of Mahometan countries, and in the beggars of all. Far be it from us to apply these facts to the explanation of the sang-froid of the magnetized, under the prick of a pin. Such an explanation would be unworthy of the subject.

composed it of the representatives of contrary opinions on the question, and of members occupied in various particular scientific pursuits. You sent both classes to the facts, because, on the one hand, whatever were their previous convictions, you had confidence in their good faith; and, on the other, by reason of the variety of their scientific tendencies, you thought they would examine the facts in all their aspects.

Gentlemen, we may at once tell you, this precaution has had in some degree its reward. With our various ideas for and against, no difference, as you will see, has arisen among us on the facts of which we have been witnesses; with our varied propensities to consider facts in particular aspects, we have been unanimous in each of our conclusions. You will find, perhaps, in this a new warrant of their truth; for it was necessary that the facts submitted to our examination should have very strong positive or negative evidence, to induce every time a constant unanimity among Commissioners always at issue on the theoretic value of animal magnetism." 954.

All sincere friends to Mesmerism must deplore the institution of this Commission. Its labours and its conclusions shew conclusively how the greatest truths may wear the semblance of the grossest falsehoods. That Mesmerism is a great truth is unquestionable. Yet observation and induction, the two great roads, it is believed, to real knowledge, *seem* directly opposed to it. Now, as Mesmerism is certainly right, it follows, as a logical consequence, that observation and induction are wrong. It seems impossible that both can be true. Who can hesitate which to believe?

We leave these exhibitions of human weakness and of human obstinacy, and turn to the grateful task of gently examining the principles, and glancing at the details of this science, more mysterious and sublime than any other, saving, perhaps, astrology.

The first wish that naturally occurs to the mind of an inquisitive person, is to learn the mode in which effects so wonderful as those of animal magnetism are obtained. He hears that the laws of nature are reversed—that the mere caprice of one individual arrests sensation and voluntary motion in another—that at the mental bidding of A the brain of B shall cease to think, to feel, to will—that the same B acquires a power, supposed to be denied since the age of miracles, of foreseeing future events—that the elaborate apparatus of vision, that exquisite contrivance, becomes altogether unnecessary, and B can see with the hair of her occiput, or read through her clothes, stays, busk, and all, with her epigastrium, or her navel. He is told that the laws of Nature and of God are set aside, and he trembles to think that the arguments for God's existence, drawn from contrivance and design, are bootless, because the same effects are now obtained without that mechanism by which contrivance and design were proved. His astonishment at this, perhaps his dismay, will be heightened when he finds by what simple means these great results have been obtained, when he perceives that he has only to turn his face one way, and to put his thumb another, to revolutionise Nature. This is another proof of the simplicity of the scheme of Providence, and our only wonder is, that since it is so easy to see without eyes, and to ascertain future events, so much trouble should have been taken in the manufacture of the former, and people should experience such unaccountable difficulty in determining the latter with precision. Mr. Murphy must be magnetised before he publishes his next year's almanack.

We have said that the means and the mode of developing the phenomena of magnetism are simple. The facility with which they are excited is singular.

Mesmer, who must be admitted to have produced the most marvellous effects, thus describes, in a series of questions and answers, the manipulations which he found to answer the purpose.

" Q.—How are the effects of the animal fluid demonstrated?

" A.—When a healthy person is brought into immediate contact with a sick person, in whom one or more functions are disordered, the latter feels, in the morbid parts, sensations more or less acute.

Q.—How must a patient be touched to make him feel the effects of magnetism?

A.—You must place yourself opposite to him, with your back turned towards the north, and draw your own close against his feet; you must then place, without pressure, both your thumbs on the plexus of the nerves in the epigastrium, and stretch your fingers towards the hypochondria. It is beneficial occasionally to move your fingers on the sides, and especially in the region of the spleen. After having continued this exercise for about a quarter of an hour, you should change your mode of operating, according to the state of the patient.

Q.—What ought to be done before we cease magnetising?

A.—You must endeavour to put the magnetic fluid in equilibrium in every part of the body. This may be done by presenting the index finger of the right hand at the summit of the head on the left side, and then drawing it down the face to the breast and over the lower extremities. In this manœuvre an iron rod may be used instead of the finger." 140.

The directions are precise, the "fingering," to use a musical term, easy, the consequences great. How often must honest folks have magnetised one another, without knowing it. Many a physician and surgeon must have had his back turned to the North, and, while kneading some abdominal tumor, done all, or more than all, that Mesmer directs; unconscious all the time of the somnambulism and the convulsions, the prophecies and the visions, he was stirring up. There cannot be a more striking proof of the blindness of medical men, in general, than their universal ignorance of the fact disclosed by Mesmer, that, when a healthy person is brought into contact with a sick, the latter feels sensations in his peccant part. From the days of Hippocrates to these, no medical author has mentioned that. Yet what an important agent it must be in diagnosis. If the mistress is sick, all we have to do is to order up the cook, bring them into immediate contact, and discover madam's weak points at once.

But even magnetism did not start into existence like Minerva, perfect and mature. The Marquis de Puységur dispensed with some manœuvres, and introduced others. The Marquis was a generous man. His fingers were moved only for the good of humanity. Like kind old Isaac Walton, who directs us, when putting a live frog upon the hook, to "treat him tenderly as we would a brother," the Marquis teaches us to magnetise ever philanthropically.

" You are," quoth the Marquis, " to consider yourself as a magnet; your arms, and particularly your hands, being its poles; and, when you touch a patient by laying one of your hands on his back and the other in direct opposition upon his stomach, you are to imagine that the magnetic fluid has a tendency to circulate from one hand to the other through the body of the patient. You may vary this position by placing one hand upon the head and the other upon the stomach, still with the same intention, the same desire of doing good. The circulation from one hand to the other will continue, the head and stomach being the parts of the body where the greatest number of nerves converge, these are, therefore, the two centres to which your action ought to be mostly directed. Friction is quite unnecessary; it is sufficient to touch with great attention, endeavouring to produce an increase of heat in the palms of the hands, &c. All the magnetic effects are more or less beneficial; one of the most satisfactory is somnambulism, but it is not the most frequent, and some patients may be perfectly cured without entering into this particular state. We should not always be intent upon producing somnambulism, for the desire of obtaining any particular result is frequently the cause of no effect whatever being produced. No magnetiser should blindly rely upon nature for the proper regulation and control of the effects which he expects will result from his magnetic operation."

M. de Puységur further adds, "If, when magnetising a patient, you perceive that he experiences a certain numbness, or slight spasm, attended with nervous shocks; and should you then observe that he closes his eyes, you must rub them lightly with your thumbs to prevent the convulsive winking of the eyelids. You will know that your patient is in the magnetic sleep when you see him sensible to your action when you hold your thumb opposite to the plexus. A patient, in his crisis, should answer no one but his magnetiser, and allow nobody else to touch him."

The somnambulic state requires the greatest caution. Man in the magnetic state is to be considered as the most interesting being in existence. With regard to his magnetiser, it is through his unbounded confidence in you that you have been enabled to bring him completely under your control. It is, therefore, for no other purpose but that of benefiting him, that you have any right to exert your power. Attempting to deceive him, or abuse his confidence, while in this state, is to commit a dishonest action, having a tendency contrary to his benefit." 144.

How beautiful is the idea of placing one hand on a man's head and another on his stomach, from pure benevolence. We suppose it is the contrary motive, rather than the blow, which makes what is technically called "a double up," so distressing. We quite agree with the Marquis, that "man in the magnetic state is the most interesting being in existence." The next most interesting creature is the magnetiser. Can any more touching exhibition be imagined, than a friend of humanity, with one hand on the occiput and another on the stomach of a fellow-creature, setting him asleep or throwing him into convulsions from the mere exuberance of his philanthropy. Perhaps the magnetiser, like the knife-grinder's friend, prefers this philosophical charity to the coarse and vulgar sympathy of common minds, and, if asked for a substitute for his magnetic benefits in the shape of some small coin, would reply :—

"I give thee sixpence, I would see thee damn'd first!
Sordid, unfeeling, reprobate, degraded,
Spiritless outcast!"

But the Marquis was not doomed to stop here. The progress of genuine discovery is slow. We have seen the virtues of putting one hand on a person's head, and applying the other to his stomach. Accident, the parent of so many inventions, stood god-father to one of the Marquis de Puysegur's. According to his—

"Usual habit of an afternoon,"

"I was magnetising," says he, "a young man by laying one of my hands on his head, and the other on his stomach. After a quarter of an hour's attention and concentration on my part, and perfect tranquillity on his, he told me that he felt nothing. As he had no complaint, this appeared to me quite natural. I however again pressed him between both my hands, merely to try whether I would be more successful; but he felt no more this second time than he had the first. I was at length about to leave him, when, on slowly removing my hand from his stomach, he fetched a sigh, and complained that I was hurting him. As I did not then touch him, I could not at first believe it; but he hastily took my hand and lowered it, saying that it stopped his breath. I quickly brought myself into contact with him, expecting that he would now feel a more decided sensation, but it proved quite the contrary; the pressure of my hand had no effect whatever. On removing it to a distance of about one foot from him, he again complained; at two feet distance, he felt a weight on his breast, and desired me to withdraw. I then drew myself back by degrees, and stopped only when he told me his pain was gone and he felt nothing. I was then five paces from him; I magnetised him at that distance by a slow and circular oscillation of my hand; and immediately his head reclined on his shoulder, and somnambulism supervened." 148.

The consequence of this remarkable fact was, that M. de Puységur ever after magnetised at a distance. In his last memoir, he asserts that a great number of facts left no doubt on his mind of the superior advantage of this new mode of operating. We speak with submission, not being just now en rapport, but we think that the Marquis was right. We feel confident that, whether he stood near or at a distance, whether his hands were on his patient's pole or on his own, it was the same. The Marquis was so good a man, and had such a fund of magnetic virtue, that he could have magnetised a post, or through one.

So far as we have gone, we have seen simplification on the increase. Mesmer had an apparatus of tubs and bottles, carried a wand, played on the piano, and wore flesh-coloured silk inexpressibles. The Marquis de Puységur dropped the tub, the wand, the breeches, and the music—and was content to manipulate his patient's stomach. Even this was abandoned for gestures at a distance, which the Marquis found more powerful than all. If he produced such effects across a room, what would he have done a mile off! They would have increased, beyond question, in a geometrical ratio. But differences still arose among the magnetisers.

"Some maintained that gestures performed without the concurrence of the will to act, and even when accompanied by a contrary volition, were nevertheless perfectly magnetic, and actually produced the usual effects. Others, on the contrary, insisted that the will to act should always accompany the gestures; and that these without the will were absolutely powerless. There were even some who looked upon the gestures as a useless accompaniment to the operation, and saw nothing more in them than a mechanical contrivance, calculated to fix the attention and sustain the will of the magnetiser, the latter being considered as the sole cause of the magnetic phenomena." 146.

Thus some believe that the will does every thing, and some that it is the gestures. We are much in the situation of Zadig. One sect at Babylon contended that salvation could only be obtained by hopping into the temple of Baal on the right leg. Another sect assured of destruction all who did not hop in upon the left. Zadig jumped in upon both. As one batch of magnetisers tells us that the will does nothing, and as another informs us that gestures do nothing, we humbly propose, as pacificators, that neither one nor the other does any thing. However this may be, M. Dupotet is not quite sure that magnetism at a distance answers, and he cites and approves the directions of M. Deleuze for the magnetic process. He alludes, en passant, to the suppositions, that the magnetiser, in order to be successful, should possess the cardinal virtues of faith, hope, and charity; and that magnetism can take no effect in the presence of a sceptic. M. Dupotet himself does not coincide with these views. But we were alluding to the directions of M. Deleuze. Here they are.

"Having thus conferred together, and resolved upon treating the subject seriously, remove from the patient all such persons as might annoy you, and keep none but the necessary witnesses (one only, if possible,) in the room. Then prepare yourself so as to be neither too warm nor too cold, and to enjoy perfect freedom in your gestures; you should also take your precautions not to be interrupted during the sitting. These preliminaries arranged, seat your patient as conveniently as possible, and place yourself opposite to him, on a seat rather more elevated than his, so as to hold his knees between yours, and to touch your feet with his own. Request him to give himself up, to think of nothing, and not to distract his attention by examining the effects he may experience; to be full of hope, and not to be uneasy or alarmed, should the magnetic influence produce in him momentary pains. After having composed yourself, hold his thumbs between your fingers, so that the inside of your thumbs may touch the inside of his, and fix your eyes upon him. You may remain from two to five minutes in this position, or until you feel that your thumbs and his are at the same temperature. This being done, you must withdraw your hands, by moving them outwardly right and left, so that the inward surface be turned outwards, and raise them as high as the head; you must then lay them on both shoulders, and leave them there for about one minute; then bring them down along the arms to the extremity of the fingers, touching slightly all the way. You will repeat this manipulation five or six times, keeping your hands off the body when you raise them. You will then hold your hands above the head for a moment, and draw them down before the face, at a distance of about two inches, as low as the pit of the stomach. Here you will stop again for about two minutes, laying your thumbs on the pit of the stomach, and your fingers under the ribs. You will then slowly come down the body as low as the knees. These manipulations should be repeated during the greater part of the sitting. You will also occasionally come nearer to the patient, so as to lay your hands behind his shoulders, and bring them slowly down the spine, and thence over the hips and along the thighs, down to the knees or to the feet. When you wish to bring the sitting to a close, you must take care to draw the magnetic fluid to the extremities of the hands and feet, by lengthening your line of motion beyond these extremities, each time shaking your fingers. Lastly, you will make before the face, and even before the breast, a few transverse manipulations, at a distance of three or four inches. It is essential to magnetise invariably downward from the head toward the extremities, and never upwards from the extremities toward the head. The downward manipulations are magnetic,—that is, they are accompanied with the intention of magnetising. The movements made upwards are not so." 151.

There are some of these arrangements which must receive our approbation, and some which must excite astonishment. We cannot but admire the wisdom of removing all persons that might annoy the magnetiser, inquisitive people who suspect delusion or fraud. The advice to the magnetiser to keep himself neither too hot nor too cold, in other words to make himself comfortable, is judicious. It is judicious too, to urge the patient to give himself up, and not to examine the effects he may experience. A vivid curiosity is beneficial in other sciences, but much to be reprobated in animal magnetism. Faith is a magnetic virtue. The manipulations enjoined by M. Deleuze are remarkable. That gentleman seems to act by a rule we had often heard of, but never seen so circumstantially set forth—the "rule of thumb." And, if the proof of the pudding be in the eating, a very excellent rule this must be. Thumb a man downwards and he sees with his rump, or is at once a ready-made prophet—thumb him up again, his bum returns to its old ignoble offices, and he knows no more of what will happen this day twelvemonths than ourselves. Wonderful science!

But there is, says M. Dupotet, a great obstacle to the development of the magnetic power—pride. "One of my somnambulists once observed, that a man may possess an independent fortune, which may allow of his time being devoted to the relief of the poor, to which he may add all the conditions of disinterestedness, persevering application, strength, charity, &c.; and yet he may not be entirely successful, because in his heart the pride of success unnerves his faculty of doing good." Incomprehensible, as well as wonderful science!

M. Dupotet warns us against premature exclamations of "extraordinary," "impossible." We agree with M. Dupotet. The proselyte of Mesmerism should deem few things extraordinary, and none impossible. But let us examine the physical phenomena.

"The symptoms most commonly induced are the following:—slight pricking and winking of the eyelids—an increase, or perhaps diminution, in the pulsations of the heart—a sensible alteration in the temperature of the body—the cheeks sometimes are flushed, or become extremely pale—the expression of the countenance, indeed, undergoes a remarkable change—stretchings of the limbs and deep yawnings succeed—a gurgling noise (borborygmus) is often heard in the throat—the patient is, perhaps, disposed to move, yet feels unable to do so, or experiences an unusual sense of composure, which is to him a peculiar, an undefined delight—the breathing frequently becomes much affected, and by a singular anomaly, occasionally the circulation increases in rapidity, while the respiratory movements of the chest become less and less frequent. In one case, particularly, which fell under my observation, the pulse previous to the operation was sixty-five, the inspirations twenty-four per minute; after the operation, however, the pulse rose to one hundred and fifteen, and one hundred and twenty, while the inspirations fell to twelve. These are the primary and most simple effects of animal magnetism; but often, under circumstances which it is previously impossible to determine, phenomena of a more remarkable character are developed. The eyelids of the magnetisee appear spasmodically affected, and close against his will; in vain does he attempt to open them, or change his attitude, in order to keep himself awake; for, if the magnetiser persevere, he yields gradually to his influence, and sleep, more or less profound, supervenes. His head, by its own weight, inclines forward upon the chest, or more rarely, is thrown backwards; his eyelids are generally half-open, and the eyeball moves slowly in the socket; its motions may be followed by the observer, who will perceive it gradually become fixed, drops of mucus fall from the lips, the limbs become cold, and the respiration audible. If spoken to, the magnetic sleeper may perhaps attempt an answer, and appear manifestly unable to speak, or he will suddenly awake, rub his eyes, stare round him with astonishment, and recollect what has passed, as we may recal a dream." 33.

This magnetic slumber seems to us to have been described by anticipation by Pope in the Dunciad. A prosy orator reads to the assembled dunces.

Soft creeping words on words the sense compose,
At every line they stretch, they yawn, they doze.
And now to this side, now to that they nod,
As verse or prose infuse the drowsy God.
Who sat the nearest, by the words o'ercome,
Slept first; the distant nodded to the hum.
Then down are roll'd the books; stretch'd o'er 'em lies
Each gentle clerk, and muttering seals his eyes.
As what a Dutchman plumps into the lakes,
One circle first, and then a second makes;
What dulness dropt among her sons imprest
Like motion from one circle to the rest:
So from the midmost the nutation spreads,
Round and more round, o'er all the sea of heads.

It must be obvious that this general somnolency was not from the gentle dulness of the orator. It was clearly magnetic, and on magnetic principles should be explained. The orator's gesticulations, his thumbing of the pages, perhaps his occasional blowing of the nose, were mines of inexhaustible, though then unknown power, and sent his audience to sleep.

But the sleep of Nourjahad himself is hardly comparable to that of the "magnetisees." M. Dupotet cites a number of the best attested cases, in which the individuals slept so soundly that they were pricked and burnt, had fingers put in their eyes, and heard pocket-pistols suddenly let off in immediate contact with their ears, yet manifested no sign of suffering, surprise, or consciousness. Take the following fact, one of the shortest, but, perhaps, one of the weakest, as a specimen.

The tenth volume of the Bibliothèque de Médecine contains the memoir of a female somnambulist, who was insensible to the lashes of a whip inflicted on her bare shoulders; and once she had her back well besmeared with honey, and in this state was exposed to the stinging of bees, under a scorching sun, yet although severely blistered, she did not manifest any sign of pain until she was awakened, when she suffered acute agony, and complained grievously of the cruel treatment she had experienced.

It is a pity that Regulus could not be magnetised. Fortunately for humanity, the pain of operations may be said to be at an end. M. Dupotet relates cases in which moxas were applied, abscesses opened, and tumors cut out, without the patient knowing any thing of what was going on. It would be the acmé of cruelty and injustice to operate on any one not made insensible by Mesmerism.

It is disagreeable, if not unphilosophical, to seek simple explanations for marvellous phenomena. The mind loves to repose on wonders, and is annoyed at the scepticism which debases by bringing them to the level of the ordinary operations of the understanding. A contemporary writer on animal magnetism,* whose statements we have already quoted, has endeavoured, and with apparent success, to shew that this insensibility to irritants or injuries is common, independently of magnetism.

Nay, the letter-writer goes so far as to produce a tally to M. Cloquet's

* Medical Gazette, March 17, 1838.

case, a thing which must be considered particularly unkind of him, because the Mesmerists make that case their cheval de bataille.

"Our case," writes the correspondent of our contemporary, "is this; it occurred some few years since, and, for obvious reasons, the names are suppressed:—A young lady, subject to hysterical fits, fell down in one of them, and struck her forehead; she was taken up quite insensible, with her features fixed, her pulse regular, her respiration easy; in short, in a state of deep sleep—of trance. There was a puffy tumor over the situation of the blow, and under the idea that injury might have resulted to the brain or its membranes from the violence, she was trephined after having been insensible for two days. The whole of this painful operation elicited from her *no expression of sensibility;* every thing was found healthy, and the wound was closed again: she remained for two days more in the same state, and then awoke in her usual health. The wound did well, and, with the exception of one or two more similar fits, she experienced no further inconvenience than before the accident, her hysteria being soon after completely cured. On awaking, she detailed all the proceedings of the operation of which she had been *painlessly sensible.* In this particular alone did the case differ, as regarded sensibility, from M. Cloquet's, and from those before related of Sauvages, Lorry, &c., as well as from those of the fanatics of St. Medard, whose histories we shall take occasion again to refer to at length, who endured, apparently without pain, and even called for, the most violent blows from heavy weapons on the most sensitive parts; and Bodin, in his Demonomanæ, p. 164, gives insensibility of parts of the body as one of the most frequent signs of *possession.*"

A moderate acquaintance with the history of enthusiasm, with the tricks of imposture, and with the phenomena of hysteria, would lead reflecting persons to the conclusion, that strange as the effects attributed to Mesmerism may appear, they are explicable on the supposition of the influence of some or of all those agencies. But such a conclusion would imply the exercise of judgment, the institution of comparisons, the calculations of reason, and the operations of knowledge. A tedious process, disgusting to some, unintelligible to others, and not half so acceptable to the mass of men, as the instantaneous action of a mysterious dogma. The recipe for successful quackery will probably always answer:—"Tell men what common sense says is impossible."

It cannot be a matter of astonishment that convulsions should be excited, as well as somnolency. An agent so powerful as animal magnetism may well produce such an effect. It is no joke pointing a finger at some patients, at least M. Petit found it none. M. Dupotet first directed his hand towards the right wrist, which immediately became convulsed; he then stood behind the patient and directed his finger first towards the left thigh, then towards the left elbow, and lastly, towards the head. Each of these parts was almost immediately seized with convulsive movements. But bad as it is to have a magnetic finger turned to us, it is ten times worse when it comes to toes. M. Dupotet, for instance, directed his left leg against that of the patient, which became immediately so much agitated that he nearly fell off his seat. He then directed his foot towards the right elbow of M. Petit, which became violently agitated; he then stretched his foot towards the left hand and elbow, and violent convulsive movements developed themselves in the upper limbs. If M. Dupotet had actually given M. Petit a kick, the consequences must have been dreadful. Imagine a magnetiser kicking one down stairs!

It is unfortunate, because it generates scepticism, that the convulsions of hysteria, and of nervous temperaments, are so similar to those produced by Mesmerism as not to be distinguishable from them. We need scarcely cite instances in point. Who has not seen a tone, a gesture, favour or interfere with the paroxysms of hysteria? Who ever dreamt of the operation of any occult agency? Surely the Mesmerists deserve great credit for investing even familiar phenomena with the garb of mystery and with the interest of unintelligibility. And it must be recollected that they have the advantage of physicians and surgeons in general. If the winks and nods of the mere doctors affect a nervous female, it is only when she can see them. But the magnetisees see as well with the back as the front of their heads, and a gesture that is out of sight will affect them as much as one that is in. Yet M. Berna's experiments shew that it is highly desirable for the magnetiser and the magnetisee to understand each other perfectly, and to proceed by fixed and regular rules. Accidents are then less likely to happen, and the convulsions, or the somnolency, occurring in the proper place, and according to the proper routine, the science of Mesmerism is properly appreciated.

Imitation has been vulgarly supposed to have much to do with the convulsions, and with the other phenomena of Mesmerism. Analogy, indeed, and reason would favour that idea, but reason and analogy are fallacious.

"It is but a short time," says the correspondent of the Gazette, "since we saw nearly every woman in a large surgical ward in hysterics, the example for which had been set by one who saw a slight operation performed on another patient. Dr. Bright mentions a similar case. Dr. Darwin speaks of a number of nuns who were successively afflicted with hysteria, and amongst other symptoms, an anxiety to imitate the squalling of cats. Dr. Whytt describes a disease common in the Island of Zetland, which affected a number of young unmarried women, and some few of the opposite sex. There were violent palpitations, convulsions of the limbs, and difficult respiration. If one was seized in the market, or at church, all who had ever been affected before directly followed the leader, and each such general disturbance was sure to increase the number of convulsionnaires. Boerhaave had a similar case in a number of boys and girls in a charity-school at Haerlem, and Dr. Haygarth another in Anglesey, where in two or three months 18 girls were affected with pains in the head and side, convulsions, and other symptoms, which magnetisers would have called magnetic. In short, the acquirement of even severe hysterical affections, if not of epilepsy and other nervous disorders, by imitation, is well known, and its occurrence guarded against by every practical physician, and even by the public."

This wears a specious air. But why trouble ourselves to weigh the effects of imitation, when a readier and an ampler solution presents itself in the powers of magnetism?

If the physical phenomena of Mesmerism are strange, the psychical are infinitely more so. It is in the latter that the science shines in all its glory, that it exalts the intellect, confounds the senses, and shews itself superior to the laws of nature.

"In all ages," says M. Dupotet, "the human mind has been a perplexing problem for the study of philosophers, who have narrowly watched its development in health, its aberrations in sickness, and its occasional unclouding in the hour of approaching death." 58.

M. Dupotet adds in a note—

"The unclouding of the mind, previous to death, or the prevision of the dying, is a phenomenon manifestly identical with the clair-voyance, or lucidity, of the magnetic somnambulist. Thus does the study of animal magnetism, as we go deeper and deeper into its apparent mysteries, assume a peculiarly sacred interest; it is the unveiling to us of our spiritual nature, and leads us onward even to the verge of that future state of existence, which all men, as they approach, even the most shallow Pyrrhonists, contemplate with a feeling of awe, not unmingled with apprehension." 58.

Metaphysics have always been perplexing, it is true; but the perplexity has not extended beyond the mental operations. Men have never doubted that the eye was exclusively made for seeing, or the ear for hearing. But Mesmerism enjoys the proud distinction of not only rendering the obscure mental operations more obscure, but even of contradicting and confusing the clearest dictates of reason. The clair-voyance of the magnetiser is identical, says M. Dupotet, with the prevision of the dying. We think that highly probable, and the one fact is much on a level with the other. In these sceptical times, prevision is not much observed on death-beds. It has not been our fortune to hear dying persons foretel the future, nor to see them distinguish the figures on a pack of cards through a party-wall. The evidence of their *having done* so is, however, as good as that of the magnetisee's *now* doing it. Whether the interest attached to animal magnetism is "peculiarly sacred," we will not pretend to determine; but certainly it leans for support on all that is recondite, abstruse, and mysterious, and its favourite analogies are drawn from phenomena which usually provoke the smile of incredulity. The more wonderful and monstrous a story of somnambulism, the better it suits the tastes and purposes of Mesmerism. But this cuts two ways. By proving that Nature abounds in extravagances, the scepticism extended to the extravagances of magnetism is, of course, diminished; and so far it is benefited. But, on the other hand, the very prevalence of wonders of this sort, is calculated to make us suspect that the wonders of Mesmerism are of a similar description, the result of natural causes naturally explicable, and not the effects of any special agency. The unbeliever too is hardened in his unbelief. Being convinced of the fallacy of one set of facts, he is not much prepossessed in favour of the set they are brought to support.

The case, for example, reported on the authority of the Archbishop of Bordeaux, would, if duly swallowed, establish the fact that eyes are not necessary for the act of vision. Proving therefore, something marvellous and impossible, it is just the fact for the magnetizers, who are wholesale dealers in wares of the same sort. The authority of the Archbishop is consequently treated with becoming deference, and his case receives the confidence so justly due to it. Perhaps some of our readers may not be aware of the excellence of the Archbishop's story, nor of the faith that it deserves. Being short, we shall quote it.

"It is that of a young ecclesiastic who was in the habit of rising during the night, in a state of somnambulism, and writing his sermons. When he had finished one page of his manuscript, he would read what he had written aloud, and revise it. In so doing he made use of the expression "*Ce divin enfant*," and in reading over the passage he changed the word *divin* for *adorable*, and then observing that the pronoun *ce* could not correctly stand before *adorable*, he added to it the letter *t*. In order to ascertain whether he made any use of his eyes, the archbishop held a piece of pasteboard under his chin to prevent his seeing the paper before him; but he continued to write on without being at all incommoded. He copied pieces of music while in this state, during which his eyes were observed to be perfectly closed. It also happened that the words were written in too large a character, and did not stand over the corresponding notes; he soon perceived the error, blotted them out, and wrote them over again with great exactness." 62.

Mr. Burchell, in the Vicar of Wakefield, expressed his admiration of facts of this description, by the exclamation—"fudge!" But the respectability of an Archbishop must preclude the idea of his telling a lie, although his account does really look very like one. It would be inconsistent with clerical obedience, for the curate to humbug his ecclesiastical superior.

Most of our readers must have heard the Irishman's answer when asked if he was acquainted with Hebrew. "Plase your honour, I can't tell till I try." Though people may laugh at the apparent bull, it really is no bull at all. This is evident from a fact, respectfully quoted by M. Dupotet from "an English Physician, Dr. Sibley."

"'It lately happened,' says he, 'that a young gentleman, about fifteen years of age, from one of the public schools, slept in the same room with me. He chose to go to bed early, and when I came into the same apartment about two hours after, he appeared remarkably intent upon his studies though fast locked in the arms of sleep. I stood some time at his bed-side and heard him repeat several lines from Homer and Virgil, after this, he repeated, with a bold and nervous accent, the whole of the Hebrew alphabet, then turning, seemed to fall into a more composed sleep. The next morning, at breakfast, I related this circumstance to the company in the presence of the young gentleman, and all were instantly commending the great progress he had made in his studies. The young man instantly declared, that however conversant he might be with Virgil and Homer, he had never heard the Hebrew alphabet repeated, nor did he ever know the name of any one of its characters.'" 65.

The value of Dr. Sibley's testimony is enhanced by the fact of his being the author of "a Key to Physic, *and* the *Occult Sciences*." The case is related in that work, and, must have been intended, as it was admirably calculated, to support some of the "Occult Sciences" of which it treated.

It is not requisite, we think, to multiply instances of this species of fact. They abound in Continental writers, in antique chroniclers, in times and countries famous for credulity, and disposed to it from ignorance. Those who have a relish for such facts may easily cull plenty of them; those who have not, will fail to be convinced by numbers of them. Animal magnetism naturally appeals to them for analogical support. Itself a compound of wonders and impossibilities it absorbs all minor monstrous things, as Aaron's rod swallowed the rods of all the Magi.

We have said that M. de Puységur discovered magnetic somnambulism. And a discovery it was. There never was, and there never will be, any thing fit to hold a candle to it. The Priestess of Delphi, Joan of Arc, Johanna Southcote, were mere bunglers. Just listen to a fraction of M. Husson's account of a magnetic somnambule.

"The somnambulist has his eyes closed; he neither sees with his eyes, nor hears with his ears: yet he sees and hears better than a waking person. He sees and hears only those with whom he is in relation. He sees only that at which he looks; and he usually looks at those objects only to which his attention is directed. He is submissive to the will of his magnetiser in all things which cannot injure himself, and in all that does not oppose his own ideas of justice and truth. He feels the will of his magnetiser. He sees, or rather he has a perception of, the interior of his own body, and of that of others; but he usually remarks those parts only which are not in the natural state, and which disturb the harmony of it. He recals to his memory things which he had forgotten in his waking state. He has previsions and presentiments, which may be erroneous in several circumstances, and which are limited in their extent. He expresses himself with surprising facility. He is not free from vanity; his self-improvement is progressive (*il se perfectionne de lui-même*) for a certain time, if guided with discretion; but if ill-directed, he goes astray." 77.

M. Deleuze swears to all this, and to something more, for he declares positively that the somnambule "hears an intelligence, a soul speaking to him, and revealing what he wishes to know." Who would not be a somnambule?

Well may M. Dupotet say that the most extraordinary phenomenon of magnetic somnambulism is the clair-voyance, or vision without the use of eyes. Instances of this are so numerous, nay so oppressive by their numbers and their authenticity, that M. Dupotet even apologises for detailing them. We may take at random one or two of these excellent models of facts. It is detailed on the unexceptionable authority of M. Chardel.

"The somnambule having recovered her senses (for she had just been seized by syncope), called for some water; I went to take a decanter from the mantle-piece, but it was empty. I took it, for the purpose of filling it, into the dining-room, where I had observed a filtering tank; I turned the cock, but no water came; and yet the tank was full. I thought that the cock should first be unstopped, and I did it with a piece of wood which I split off; but still the water did not come out. I then supposed that the air-hole of the reservoir was obstructed, and as it was very narrow, it was necessary again to split the piece of wood in order to introduce it: but I was not more successful than before. At last I resolved upon filling my decanter with unfiltered water. My somnambule was still in the same attitude in which I left her. She had seen me all the time, had followed all my movements, and detailed them to me without omitting a single circumstance, notwithstanding there was between her and me two walls and a parlour,—and my actions included a number of minute details which nobody could have imagined." M. Chardel proceeds:—"I might adduce many more instances of similar sight, and even at considerably greater distances; but the circumstances would not be more conclusive." 88.

If this is not evidence we should like to know what is. If enabling a young woman to see through two walls and a parlour, is not conclusive in favour of magnetism, there is an end of all rational inquiry. A few facts of this description would prove anything. They are like the "*alleybi*" witnesses in the Pickwick Papers. Let us take another of these "duffers." Dr. Despine deposes:—

"Not only did our patient hear with the palm of her hands, but we saw her read, without the help of her eyes, and with the extremities of her fingers, which she rapidly agitated over the page which she intended to read, and without touching it, as if to multiply the feeling surfaces; she thus read a whole page of a novel by Montholieu.

At other times we saw her single out of a parcel containing upwards of thirty letters, one which had been pointed out to her, and read on the dial, and through the glass of a watch the hour indicated by the hands; she also wrote several letters, corrected them by a second reading, marking the mistakes as she went on, and recopied one of them, word for word. During all these operations, a thick pasteboard screen intercepted in the most effectual manner every visual ray that might have reached the eye.

The same phenomenon took place at the sole of the feet, and at the epigastrium; and the patient seemed to experience a painful sensation when simply touched." 89.

There are two or three men who have deserved and acquired great reputation for the accuracy of their observations, and the simplicity of their statements. Ferdinand Mendez Pinto, Mandeville, Munchausen, have all great merit in their way. But it seems to us that M. Chardel and Dr. Despine are very likely to eclipse it.

It is obvious that phenomena of this description are beyond reason, contrary to experience, utterly irreconcileable to common sense, and therefore to be received on some special grounds. Those grounds resolve themselves into the weight of testimony. No candid man can quarrel with that. He is only asked to disregard the whole experience of his life, to disbelieve the accumulated evidence of his senses, at the instance of men of such eminence in science as Mesmer, M. Charnel, M. Berna, and a few others. Can any thing be fairer? Away with the narrow-mindedness which higgles at impossibilities. Away with the conceit which thinks that other men can be deluded. Away with the depravity which suspects that others may juggle or lie. These are the days for universal belief. We are bigots if we laugh at homœopathy—bigots if we disbelieve animal magnetism. Each has been shewn to be opposed to reason; each has been supported by testimony. Let us hear no more of the age of reason; ours is the age of witnesses.

They have proved that a woman may see through two walls and a parlour—that a person who knew nothing of Hebrew, could accurately recite the Hebrew alphabet—that the epigastrium or the bum was just as good an organ of vision as the eye—nobody can doubt it. But this is child's play.

In order to shew that magnetic somnambules enjoy the power of divining futurity; M. Dupotet shews, first, that this is easy and has been common enough, and, secondly, he gives the most satisfactory examples in the cases of magnetisees. The faculty of prevision, he says, "has been in all ages observed, and is attested by a concurrence of evidence which is irresistible." Sceptics may laugh, but nothing can be more certain than this. We should like to know what they will say to the case we are about to cite—that of a natural somnambulist, named Arron—a fact which M. Dupotet "holds to be incontrovertibly established."

"A physician from Chartres saw her some time ago. On being introduced to her, in company with several other gentlemen, he questioned her, without being able to obtain an answer. Thinking that if he was alone with her, she might perhaps be induced to speak, he requested the spectators to withdraw. When they were both in private the following conversation took place:—

'Marie,' said he, 'do you know me?'—'Yes, sir.' 'Who am I?'—'You are a physician.' 'Whence do I come?'—'From Chartres.' 'Where is my house at Chartres?' 'In a small street running down a declivity.' 'Can you see my house?'—Yes, sir.' 'Is there any company in it?'—'Yes, sir; four ladies, one old, two middle-aged, and one young lady.' 'For what purpose have I come in this part of the country?'—'To see a female patient.' 'Where is her complaint?'—(Here she pointed to the part affected, which we cannot just now recollect.)—'Where did I dine?'—'At M.'s.' 'Was there a good dinner?'—'Yes, sir.' 'Could you tell me what dishes we had?'—'Certainly.' (She names every dish and its particular place on the table.) 'What do I hold in my hand?'—'A small wooden box.' 'What does it contain?'—'Sharp little iron-tools.' 'Now, what have I in my hand?'—'Some money.'—'How much?'—(She names the sum.) 'In what coins?'—(She specifies the various coins.) 'Can you tell me my thought at this moment?'—'Yes, sir.' 'Say it.'—'I dare not; I must not tell you.'—'Well, I will tell you: I think of giving you this money.'—'So you do, sir; but I could not say so.' All these answers were perfectly correct. Other answers no less surprising than the preceding have been reported to us, but we shall confine ourselves to these.

Sceptics (and less would suffice to provoke scepticism) will, no doubt, exclaim that it is impossible to credit such assertions; but let them inquire into the evidence, and they will then come to the conviction that we state nothing but the truth." 109.

We would ask if better evidence than that was ever produced at the Old Bailey? We would ask, what is to be said against testimony now? The followers of Mesmer are quite of old Mr. Weller's mode of thinking:—"there is nothing like an alleybi." Good, wholesome swearing for them.

This fact is aptly employed for the illustration of the prevision of magnetism. That is just the same sort of thing. But the magnetisees are in general modest, and predict, for the most part, what will happen to themselves—a fit—a delirium—or a pox—and "I have seen," says Dr. Rostan or Georget, "positively seen, and repeatedly, somnambulists foretell, several hours or days—even twenty days in advance, the precise hour and minute of the accession of epileptic or hysterical fits, and describe what would be their duration and intensity, which came to pass in exact accordance with the prediction."

We are inclined to complain of the magnetisees upon this score. While they are about it, they may as well see something good in the wind—a fall in stocks, or a revolution. We would recommend this. It would catch the practical men. There is an opening next year for an Almanack. When the patient predicts her own fit, or fever, ill-natured persons are apt to object that the verification of the prophecy is too much in her own power—that, if a cheat, she can counterfeit, and if a fool, she can work herself into the appointed paroxysm.

Exaltation of the human faculties is another, and a pleasing effect of Mesmerism. M. Dupotet, as usual, demonstrates its occurrence in sleep, and natural somnambulism. Who does not know the great things that have been done in sleep? But the mischief of it is that we forget them all on waking. Think of what the world must have lost. Animal magnetism, which puts its votaries asleep, develops, of course, these exalted faculties, which sleep, erroneously supposed a torpor of them, naturally sets agog. The magnetic somnambule conversing aloud, brings us into direct relation with that mystic essence, which is now unfettered and revelling in its freedom. Listen to its high and holy communings. The place in which they are delivered is the Theatre of the North London Hospital. There every Thursday, the interesting patients are subjected to Mesmerism, and pour out their souls in some such fashion as this.*

---

* We quote the sayings and doings of the somnambules at the North London from the Lancet.

Okey,* a girl about eighteen, loquitur, in a state of delirium.

"Oh!" she exclaimed, "what a dirty-white chair," turning it round. The chair was removed by Dr. Elliotson. She then advanced and said, with innocent familiarity, and a peculiar and agreeable tone of voice, "Oh! how do ye?" to the Marquis of Anglesea, who sat immediately in front of her. "White trowsers. Dear! you do look so tidy, you do. What nice things. You *are* a nice man." The Marquis wore bright buff trowsers. "Gloves, not white; and what a nice stick." (Turning to Sir C. Paget.) "Why do you wear your hat?"

Suppose Okey had had a natural dream, would she have recollected, should we have obtained, that exquisite idea—that "purest English undefiled"—"You *are* a nice man." But to proceed:

"Give the glass to the gentleman again," the Doctor said. "Where is he?" —"Look round and see."—"Tisn't yours, beauty-cheeks," she said, to a rosy-looking gentleman on the right. The glass restored, she proceeded to amuse herself among the throng around her. "Oh, what a beatiful oyster!"

There was a phrase—'beauty-cheeks.' But the well of her vernacular was pretty deep.

"A sheet of mill-board was placed below her chin, so that she could see nothing that was beneath it. The board provoked her displeasure, which she vented thus:—"How do you, nasty beast? Such a dirty beast you are. I don't like you. Does your mother know you're out?" (Much laughter around.) "Go along with you."

How edified the Marquises, the Earls, the Doctors, and the Feelosophers (as Mr. Cobbett calls them) must have been, at this exaltation of the intellectual faculties—at this unfolding of the very soul. The bashful, the amiable, the gifted Okey must indeed have been an interesting object when she archly enquired of the paste-board, "does your mother know you're out?" Then observe how naively she chided Dr. Elliotson.

"Don't be silly," she said, "you silly man. Oh, but you're a fool, Dr. Ellisson. Ha! ha! How do ye? Mine won't go like yours. They ain't so silly. Oh, Dr. Ellisson, leave off, its no use."

Charming Okey!

"A child was now brought into the theatre, from the wards, and placed on the chair from which Okey was removed, who, not knowing what else to do, sat down on the mill-board, and whistled and sang in a sweet tone, with once very artist-like variations, evincing symptoms of a ripe faculty of music. Ending, at last, with *Jim Crow*, she stopped in one verse to ask Dr. Elliotson if *he* also had "come over from Kentucky," like Mr. Crow, and then volunteered to "wheel about, and turn about," but was prevented, to the manifest disappointment of many spectators."

We need not quote any more of the observations of the gentle Okey, or her colleagues. What we have transcribed will amply establish the truth of M. Dupotet's remark:—"that such persons during their somnambulism often manifest a knowledge and an intellectual activity which they do not at other times possess."

Not an uninteresting feature of these exhibitions is the "rapport" that

* A descendant it is to be presumed, of the famous Okey Pokey Wanky Fum. —*Printer's Devil.*

seems established between patient and physician. The former tells the latter he's a fool, or asks him if he came over from Kentucky, with the most refreshing nonchalance. But it was for the purpose of proving the development of the mental powers insisted on by M. Dupotet, and highly characteristic in itself, that we quoted these experiments at the North London Hospital. They exhibit in a very strong point of view that singular and instructive phenomenon.

M. Dupotet justly remarks that the good preponderates over the evil in the world, and that "the current of all things flowing onwards is redolent of blessings." Certainly this is the case with Mesmerism. We have seen what that marvellous science has done. It has given to one, "tired Nature's sweet restorer, balmy sleep," and that in no niggard fashion—another, it has enabled to see through a piece of pasteboard, or two walls and a parlour, with the back of the head, the pit of the stomach, or the tips of the fingers—a third it has endowed with the gift of prophecy. If it did no more it would still, to use the beautiful language of the Baron, be "flowing onwards redolent of blessings." But the measure of its benefits to suffering humanity is very far from full. We are to see it miraculously curing the halt, drying up instantaneously the sores of scrofula, and resuscitating the dead. Even scepticism itself must blush at its suspicions of a science supported by such facts.

M. Dupotet triumphantly and properly appeals to them. Well may he boast:—"It was, I repeat, the successful treatment and cure of diseases, which had notoriously resisted every other remedy, which compelled the rudest and most inveterate of our antagonists to recognise the influence of magnetism." As all this has been proved by testimony, we see its superiority to all other evidence. The fallacious rules of logic, the ordinary operations of the understanding, are equally futile in explaining or in comprehending the phenomena of Mesmerism. But A and B swear, that what seems impossible has actually been done, and what modern philosopher would scruple to believe them?

The sceptic Hume argued in his Essay upon miracles, that it was more probable that men should combine for the purposes of falsehood, than that the laws of Nature should be reversed. But this argument lost its force when applied to the great truths of Christianity. For the interruption of the ordinary laws of Nature, for so mighty a purpose as the regeneration of the mind of man and the salvation of his soul, was less improbable than the combination of many persons, at many times, for the purposes of propagating an untruth, which, so far from being beneficial to them, must expose them, as they knew, to contumely, misery, and, perhaps, a horrible death. These considerations have induced all men of capacity and candour to reject Hume's argument as inapplicable to the case of Christianity. But they have been disposed to consider it as sound in reference to the bootless, senseless, and ridiculous miracles of modern times, to the exploits of Hohenlohe, the pretensions of Johanna Southcote, and the gift of tongues of the Irvingites. Whether it will apply to the wonders of Mesmerism, we leave to others to determine. When we extend the terms of it, and affirm, *that*, it is more probable that many persons should be deceived, and that some should deceive for the purposes of falsehood, than that the laws of Nature should be reversed, we advance a proposition that carries with it an air of plausibility, and seems to have the sanction of modern experience. Yet it must be owned that it is troublesome and disagreeable to prove and entertain it. It is far more pleasant to take things upon trust, and, as the Marquis de Puységur performed his miracles from pure philanthropy, to display the same philanthropy in the belief of them. But to the cures.

1. "A woman of forty years of age, worn out by long sufferings, and unable to stir without crutches, resolved on having recourse to animal magnetism, and was conveyed for that purpose to Paris, in a sedan-chair. She was two days travelling a distance of thirty-six miles. Several fainting fits came on during this journey. On being magnetised upon her arrival, she fell into somnambulism, but her sleep did not present any lucidity. The magnetic effects, however, produced by the magnetiser were such that he at once declared that, in a few days, the patient would walk without crutches. He therefore invited her to a ball, which he intended giving in his house on a certain day. The doubts expressed by the patient and her friends did not in the least disconcert him, but, on the contrary, he sent his invitations to a great many people, that they might go and convince themselves, and acknowledge the truth of his assertion and the triumph of magnetism. In the meantime the patient was magnetised every morning, and at each sitting a decided improvement was observed. On the eleventh day she began to attempt a few steps, being supported by the arms, and she left off using crutches. On the seventeenth, she came to the *soirée* of the magnetiser, ascended the stairs unassisted, walked about the room, and remained there until one o'clock in the morning, when she retired to her own house without having felt any more fatigue than would have been the case with a person in good health." 192.

There is a case. The petulant surgeon may possibly hint a few flaws in it. He may remark *that* the nature of the cause of the lameness is not mentioned—*that* we are left in ignorance of the character of the disorder and species of the "long suffering"—and *that* the case ends, like a novel, very happily but very suddenly. We hear of the marriage, but not of the subsequent miscarriages.

2. "Caroline Baudoin, twenty years of age, of a lymphatic temperament, had passed her childhood at Geneva, where the badness of her constitution fully developed itself, aggravated perhaps by the influence of the climate, or the use of unwholesome food. Her whole glandular system became diseased; her throat, breasts and arm-pits exhibited tumors of a decidedly scrofulous nature, many of which suppurated, and discharged an abundance of purulent matter. The disease had been treated by all the most approved means; several issues had been inserted, but other tumours gathered and burst. One in particular, in the left arm, had burrowed down to the bone, and spread through the adjacent muscles, so as to render necessary the amputation of the arm." 188.

The operation was successful, but another wound opened on her breast, and resisted every kind of medical treatment.

"Moved by the recital of her sufferings, I resolved upon magnetising her, rather from an instinctive feeling that I might relieve her, than from any conviction that I could do her good, for I scarcely considered it possible to cure so inveterate a disease. In the course of three minutes' magnetisation, she fell asleep, and began by telling me that, had she known me seven months sooner, she would not have lost her arm. It was only three months since she had been operated upon. She pointed out the means of healing the wounds on the arm and breast, and on these being applied they proved completely successful. The most important thing, however, remained to be effected, which was to change her constitution, or at least to modify it in such a manner as to prevent a recurrence of the previous eruptions. Magnetism had produced a sufficient degree of lucidity to allow of her giving advice to other patients, but hitherto not enough to describe the means of curing herself. One day, as she was prescribing for a patient whose recovery she was anxious to bring about, she interrupted the consultation, and told me that on the 24th of August, at nine in the evening, she should fall into a state of profound sleep, which would last for thirty hours; that this sleep would be very calm, if during the two preceding days she were not annoyed by anything; but otherwise she should be much agitated; and that by an unaccountable feeling she should endeavour to eat her own flesh. She therefore desired that precautions might be taken to check this fatal propensity, and requested that she might be incessantly watched. She declared further, that during this crisis of thirty hours, she would eat absolutely nothing; and that the scrofulous matter would be carried out of her system. She also said that during her sleep a *bruissement* would be heard at the epigastrium, caused by the flow of scrofulous humours. She then predicted her perfect recovery. This declaration was made on the 14th of July, 1833. I made her repeat it on the 21st of the same month, in the presence of fifteen persons, who drew up and signed a report to this effect, having previously taken care to ascertain her scrofulous state. In the intervening period many persons took cognizance of the declaration, and promised, if her prediction were fulfilled, to attest so remarkable a case. On the 24th of August, at eight in the evening, it was arranged that several persons should assemble in the house of the patient, at the Petit-Carreau; and I enjoined her attendants to put her to bed half-an-hour before the accession of her crisis, in order to prevent her being annoyed. All this was punctually done. At nine o'clock precisely, a number of visitors had congregated. On arriving, we were informed that the crisis had declared itself a few minutes sooner than she had predicted, and that it was fully developed. On entering the room we saw the unfortunate girl with her face swelled, her tongue protruding out of her mouth, nearly, to all appearance, cut in two by her teeth, her limbs stiffened, and her jaws so firmly locked that it was impossible to open them. After having magnetised the masseter muscles so as to remove the stiffness of the jaws, I caused the tongue to be drawn in, which was already very much discoloured, and fortunately had only been bitten very slightly. No one had yet perceived that one of her fingers had not only been bitten, but that there was a loss of substance, the piece wanting having been swallowed by her during her previous paroxysm. The wound was now dressed, out of which no blood, but a great quantity of red lymph issued. As the violence of this crisis continued I thought it proper to remain with her during the ensuing thirty hours. I was perfectly right in having taken this resolution, for she struggled long with extraordinary violence, and attempted to put her hand into her mouth to bite it again, but she had been so bound down that she could only get at the sheets, a piece of which she succeeded in tearing off. The somnambulic state at length terminated; her prediction was fulfilled; and she was, to the satisfaction of all the parties interested, from that day cured." 191.

If the other case was enough to " beat Bannagher," this, to follow up the analogy, must be allowed to " beat the devil." Yet cynicism, which is never satisfied, finds fault even with a cure like this. M. Dupotet remarks that, in the state of magnetisation, she pointed out the means of healing the wounds on the arms and breast, and that these means proved successful. Yet he adds that she had not enough lucidity to describe the means of curing herself. There is here an apparent contradiction. It would have been desirable to have known what the " means" pointed out and made use of were. We suppose, however, that some wounds were open on the 24th of August. It is a pity for the honour of magnetism that they were not specified. As they were " from that day" cured, it would have been useful to have been told of the process, whether granulation, or instantaneous cicatrization, or whether a new one altogether. But we pass on to a more conclusive instance of the wonderful powers of Mesmerism.

3. " In the thesis of a distinguished foreigner, M. Albert Jozwik, which was cordially received by the professors, who encouraged him to proceed in his scientific researches, several remarkable magnetical cases are reported. I extract the following,—' In the month of July, 1829, in the camp then before Warsaw, a subaltern officer of the third regiment of *Chasseurs à pied* of the Polish army, shot himself by putting the muzzle of his musket in his mouth. The medical officer of his regiment was instantly on the spot, and gave him every assistance, but in vain. This case was reported to me, as I was then superintending the medical department of the division. The body of the severely wounded officer had been conveyed to the infirmary, to which I immediately repaired; and, having found it still warm, I magnetised it. After a magnetisation of about half an hour, the poor fellow began to breathe, and was resuscitated; I then dressed his wound, and sent him to the hospital called Uiazdow." 196.

If that does not move thee, brainless sceptic, go! A revelation from Heaven itself, as M. Dupotet remarks, would not convince thee.

The Baron is of opinion, indeed he could not be otherwise, that a good work on the practice of medicine, founded on the principles of animal magnetism, is much required by medical men. Every one must lament the deficiency of a work of this description—of a work which will enable us, if we cannot treat a case with success, to throw our patient into a trance, and pump out of her the means of curing both herself and her neighbours. A singular advantage, too, accruing from this employment of Mesmerism is, that the somnambule being quite unconscious of what she has said in her somnambulism, we can, after all, take the credit of the cure to ourselves. Our readers will be delighted to learn from M. Dupotet, that—" this *desideratum* I propose endeavouring to supply in my next volume, to which this, as I have already stated, is only an introduction." We beg our country readers to be on the qui vive, and subscribe early for the valuable volume.

And yet, on consideration, it does not appear so requisite, for M. Dupotet, himself, informs us that medical science, even in its present imperfect state, is founded upon animal magnetism.

" The whole art and practice of medicine in the ancient temples consisted in the magnetic treatment which is even at the present day practised in India. Indeed, no manner of doubt can exist, that even in the hands of unskilful practitioners, in the midst of all their strange and extravagant superstitions, such men have wrought many very curious and signal cures. It is idle to suppose that any nation would believe generally in the efficacy of any therapeutical agent, were it only after all an errant folly or fiction, for however uninstructed men may be, they derive knowledge from experience, and it is not reasonable to presume that any method of treatment would for ages be persevered in, if the sick therefrom derived no description of benefit." 185.

This is a very acute argument, and when properly weighed tells no little in favour of Mesmerism. And yet on a superficial glance and in a logical form it does not seem to do so. The whole art and practice of medicine anciently consisted in magnetic treatment—the experience of men instructs them in the real value of medicinal agents—but that experience has led them to depart from the ancient magnetism, and adopt the modern facts and principles of physiology, pathology and therapeutics—therefore, magnetism is not the true foundation of physic. We merely put this in a logical form, to shew the seeming inconsistency between the Baron's premises and his conclusions. But magnetism, as it does not require, does not make use of, the ordinary usages of reasoning or reason. It is clearly superior to both.

This mystical science is not, however, exhausted. The deeper we go the deeper we may.

In the first place, the will, or magnetism, however induced, can be communicated through opaque bodies. We have spoken of the magnetic somnambule, who saw through two walls and a parlour. We will now give a crucial instance, quite as satisfactory.

"An Indian lady," says he, "residing in Paris, and possessed of great magnetic power, was solicited by a lady who accompanied me, to visit her, and give us a proof of the energy of her will. She had a maid servant, whom she kept under the magnetic treatment, and often threw into somnambulism. She was then working in a room entirely separated from us. The Indian lady, on being asked whether her maid should appear before us at her tacit mental bidding, composed herself for a moment, and magnetised her from the room where she then was sitting without speaking or making the slightest motion. A few minutes afterwards, we saw the maid-servant step into the room in which we were to inquire of her mistress what was her pleasure." 213.

What do our readers say to that? Bell-hangers beware. If we want "John" up from the kitchen, all we have to do is to compose ourselves upon our chairs, and will that John should appear. Mary will no longer say to John, "there's missus's bell," but John will quietly say to himself, "there's missus's will." The vulgar have been frequently observed to be in the advance of science, and have discovered the fact before philosophers have found out the explanation. We now see clearly, what before seemed obscure, the true nature of the common phrase—"don't you wish you may get it?" That phrase had obviously a deep magnetic meaning.

We have now displayed the principal phenomena resulting from the employment of animal magnetism. A slight analytical examination of them may not be uninstructive.

*a.* In the first place, we may remark that nothing is effected by the agency of Mesmerism, which the magnetisers themselves do not proclaim to have happened again and again independently of its assistance. The sceptic suspects, perhaps unjustly, the occasion of this strange anxiety to underrate their art. He sees, or thinks he sees, that, ashamed of its monstrosities, and alarmed at its absurdities, they are willing to blind the public eye with the dust of like absurdities and like monstrosities. Be that as it may, we observe in this respect a singular analogy between Mesmerism and Astrology. The proselytes to both believe—

> "That in this world there's not a wart
> That has not there a counter part;
> Nor can there on the face of ground,
> An individual beard be found,
> That has not in that foreign nation,
> A fellow of the self-same fashion;
> So cut, so coloured, and so curled,
> As those are in the inferior world."

*b.* Although the phenomena of Mesmerism are all wonderful, they admit of being resolved into two distinct sets. The first are simply wonderful—the second simply impossible.

The extraordinary phenomena, however extraordinary, are still not contrary to reason. If the magnetisee falls into a species of trance, raves in delirium, or is agitated by convulsions—nay, if, like the Priestess of Delphi in former times, and the Irvingite fanatics in our own, she affects to foresee and to foretel the future,—there is nothing which might not readily be generated from a heated imagination, acted on by *any* powerful impression. The very pains which are taken by the magnetisers to insist on the frequency of such phenomena, independently of magnetism, prove, if they prove any thing, that it is the nervous system of the individual acted on, and not any special power in the agent, which gives those phenomena birth. It is true that they now appear in a monstrous and exaggerated form. But the collateral circumstances are monstrous and exaggerated too. If our ordinary hysterical patients do not " go the whole hog" like Okey, their antics, it must be remembered, are *checked*, not *encouraged*. Give hysteria licence—pat it on the back—exhibit it—and offer the premium that the magnetisers do offer, on its fancies, follies, and cheats, and a thousand Okeys would soon be in the field. But if Okey received her deserts, and had a pailful of cold water emptied on her once or twice, as many an honester woman has had, we should soon find that brooches would not be taken for oysters, and her vulgar slang would be kept in the pure recesses of her own bosom, not poured into the ears of credulous marquises and gaping dukes.

*c.* The clair-voyance, the transposition of the senses, the prevision, are—" Fudge." Those who can believe any or every thing, will probably believe them. The patrons of quackery, and they are many—the proselytes of homœopathy, and they *were* a few—the lovers of the marvellous in all its forms, from the man in the bottle to the magnetic somnambule—the thirsty hunters for novelty—all these will credit the possibility and the fact, of God permitting his demonstrable and eternal laws to be reversed, on the most ridiculous occasions, for the most ridiculous of purposes, by the most ridiculous of means. They will believe that any impudent strumpet may get a permit from Heaven to exhibit at half-a-crown a head, vision with her fingers or her toes, and may be allowed to read the mystic scroll of the future, which God has wisely hid from the penetrating gaze of science, and the humble wish of virtue. It would indeed appear that—

> The pleasure is as great
> Of being cheated as to cheat.

They who can credit such gross delusions are beyond the reach of argument. Ridicule is the only method of correction. If they feel it, there are hopes. If callous to it, they would do well to " purge and let blood" when the moon's at full.

It would be hard to predict how long this epidemical folly will last. As the dog-days are approaching, it will rage, of course, for a while longer. It will count its votaries till some new absurdity, fresher, at all events, if not more monstrous still, appears on the horizon. The stock of credulity in this world is inexhaustible. Reason, experience, are opposed to it in vain. The wonder-monger, like the gambler, plays deeper the more he has lost. The next throw, he may win.

